# Reduced DPP9 levels sensitize experimental breast tumors to combinatory treatment with irradiation and Olaparib

**DOI:** 10.3389/fonc.2026.1845048

**Published:** 2026-06-10

**Authors:** Lisa Heß, Jule Koch, Finja Göttsche, Thomas Reinheckel

**Affiliations:** 1 Institute of Molecular Medicine and Cell Research, Faculty of Medicine, University of Freiburg, Freiburg, Germany; 2 German Cancer Consortium (DKTK), Partner Site Freiburg, a partnership between DKFZ and University Medical Center Freiburg, Heidelberg, Germany

**Keywords:** breast cancer, combination therapy, irradiation, Olaparib, protease, synthetic lethality

## Abstract

Breast cancer is a heterogenous disease with various molecular subtypes. Among these, triple-negative breast cancer has the worst prognosis due to its high aggressiveness and limited availability of targeted therapies. In breast cancers, reduced expression of dipeptidyl peptidase 9 (DPP9) is associated with a poor patient prognosis. To model reduced DPP9 expression, we transplanted the human triple-negative breast cancer cell line MDA.MB.231 with an inducible genetic DPP9 deficiency in the mammary fat pad of immunocompromised mice. As expected, tumors with DPP9 deficiency showed an increased weight as well as more lung metastasis compared to controls. This phenotype seems to be promoted by increased vessel formation in the tumor due to DPP9 deficiency. Upon irradiation (2 × 9 Gy), tumor growth was initially reduced independent of DPP9 expression, although DPP9-deficient tumors grew out faster after irradiation compared to controls. Additionally, more metastases were formed in mice with DPP9-deficient tumors compared to controls as well as untreated mice with tumors of both genotypes. As proteolytic cleavage of BRCA2 by DPP9 was previously shown to promote repair of DNA double-strand breaks by homologous recombination, the poly-ADP-ribose-polymerase (PARP) inhibitor Olaparib (25 mg/kg) was applied to mice in combination with local irradiation in order to test for synthetic lethality effects. The results revealed further reduction in tumor growth compared to untreated and irradiated mice. Furthermore, DPP9 deficiency together with combined irradiation/PARP inhibition further reduced tumor growth compared to control tumors. Yet, metastasis formation presented with a mixed outcome in mice with DPP9-deficient tumors. In summary, reduced DPP9 levels enhanced primary tumor growth but sensitized triple-negative breast tumors to a combination of irradiation and Olaparib.

## 1 Introduction

Breast cancer is a heterogenous disease divided into four main molecular subtypes: Luminal A, luminal B, human epidermal growth factor receptor 2 (HER2)-enriched, and triple-negative/basal-like (1, 2). Luminal breast cancers express hormone receptors like estrogen and progesterone receptors and are less proliferative. The HER2-enriched subtype is characterized by an amplification of HER2, which results in fast and uncontrolled tumor growth. The most aggressive subtype represents triple-negative breast cancer (TNBC), which includes all cancers not expressing any of the three afore mentioned receptors (3). Because of the expression of certain receptors in most of the breast cancer subtypes, treatment targeting these receptors is very promising (1). However, for TNBCs, no targeted treatment is available, limiting therapy options mostly to non-targeted approaches like surgery, irradiation, or chemotherapy (3).

By now, various proteins have been identified to influence the growth as well as metastasis formation of breast cancer (1). For instance, low levels of dipeptidyl peptidase 9 (DPP9), a member of the DPPIV family of proteases, were associated with poor overall survival (4, 5). DPP9 is an aminopeptidase cleaving dipeptides, if the second amino acid is a proline or alanine (6–8). It was shown that DPP9 deficiency in a luminal breast cancer mouse model (MMTV-PyMT model) delays tumor onset but enhances metastasis formation (4). Here, low levels of DPP9 resulted in the higher expression of the epithelial–mesenchymal transition (EMT) transcription factor ZEB1, reducing the expression of epithelial markers as well as enhancing the expression of mesenchymal markers, leading to a more migratory and invasive behavior of breast tumor cells. Furthermore, low nutrient conditions in the luminal breast cancer cell line MCF-7 led to defective autophagy (9). Targeted treatment of MCF-7 cells with 4-hydroxytamoxifen, inhibiting the estrogen receptor highly expressed in luminal breast cancer subtypes, resulted in more cell death in DPP9-deficient cells compared to controls.

Although much is known about the role of DPP9 in luminal breast cancers, there is only limited information about DPP9 in TNBCs. In patients with breast cancer, TNBCs present with the lowest DPP9 expression compared to all other subtypes and normal breast tissue (4). Therefore, we aimed to investigate the impact of DPP9 in TNBC by orthotopic transplantation (OT) of the TNBC cell line MDA.MB.231. Here, DPP9 deficiency led to a higher tumor weight as well as more metastasis formation. This seemed to be promoted by the formation of more blood vessels within the tumor upon DPP9 deficiency during early tumorigenesis.

The strong tumor growth of DPP9-deficient tumors raised the question whether low DPP9 expression already shown for patients with TNBC (4) can be utilized to reduce tumor growth. Interestingly, it was reported previously that DPP9 promotes homologous recombination (HR) by cleaving BRCA2 and targeting it for degradation, increasing the viability of cells upon DNA damage induction *in vitro* (10). Therefore, we assumed that DPP9 deficiency might result in cell death upon DNA damage induction, for instance, by irradiation. Although irradiation reduced tumor growth independent of DPP9 expression levels, mice with DPP9-deficient tumors presented still with higher tumor weight and more metastasis compared to irradiated controls. However, patients with HR defects were shown to be sensitive to poly-ADP-ribose-polymerase (PARP) inhibition. Therefore, we combined irradiation with PARP inhibition by Olaparib and demonstrated a reduced tumor growth of DPP9-deficient tumors in comparison to controls as well as all other conditions tested.

## 2 Materials and methods

### 2.1 Cell lines

MDA.MB.231 and MDA.Mb.468 cells were cultured in DMEM-high glucose, pyruvate (Gibco) supplemented with 10% fetal bovine serum (FBS: PAN Biotech), 1% L-glutamine (Gibco), and 1% penicillin/streptomycin (Gibco) at 37 °C and 5% CO_2_. HCC38 and HCC1806 cells were cultured in RPMI (Gibco), with 10% FBS, 1% L-glutamine, 1 mM sodium pyruvate (Gibco), 4.5 g/L glucose (Gibco), 10 mM HEPES (Pan Biotech), and 1% penicillin/streptomycin at 37 °C and 5% CO_2_. Human umbilical vein endothelial cells (HUVECs) were cultured in EGM-2 medium (EGM-2 Endothelial Cell Growth Medium BulletKit, Lonza) at 37 °C and 5% CO_2_.

Non- (*Ren*), *DPP8*-, or *DPP9*-targeting shRNAs were cloned and transfected in MDA.MB.231 cells as described in (9). Shortly, the lentiviral vector system pMSCV-rtTA3-IRES-EcoReceptor-PGK-Puro vector was integrated for ecotropic retroviral transduction of pTCEBAC/pTREBAV-single shRNAs into MDA.MB.231 cells. For this purpose, Plat-E cells were transfected with 3.25 μg of shRNA and 0.75 μg of p-Super-DGCR8 in 500 μL of pre-warmed Opti-MEM with 12 μL of polyethylenimine (PEI). After 72 h, retroviral particles were harvested from the Plat-E supernatant using a disposable syringe and a 0.45-μm sterile filter. For transfection, the supernatant was diluted at 1:6 in DMEM with 8 μg/mL polybrene and transferred on MDA.MB.231 cells. On the next day, medium was exchanged and selection with blasticidin (26 μg/ml) was started, which was maintained for 2 weeks. The non-targeting shRNA is binding to the Renilla Luciferase (*shRen*) not present in mammals. Sequences of the shRNAs are shown in **Table 1**.

**Table 1:** Sequences of shRNAs in miR-E backbone.

Target	Sequence 5′ to 3′
*None (Ren)*	TGCTGTTGACAGTGAGCGCAGGAATTATAATGCTTATCTATAGTGAAG CCACAGATGTATAGATAAGCATTATAATTCCTATGCCTACTGCCTCGGA
*DPP8*	TGCTGTTGACAGTGAGCGCACGGTTTGTGGTAGTAATCTATAGTGAAG CCACAGATGTATAGATTACTACCACAAACCGTATGCCTACTGCCTCGGA
*DPP9*	TGCTGTTGACAGTGAGCGCCCACGGCTTCCTGGACGAAAATAGTGAAG CCACAGATGTATTTTCGTCCAGGAAGCCGTGGATGCCTACTGCCTCGGA

### 2.2 Treatment of cells

To induce shRNA expression *in vitro*, MDA.MB.231 cells were treated with 2 µg/mL Doxycycline (Sigma). Inhibition of DPP8 and DPP9 was achieved by treatment of cells with 10 µM 1G244 for at least 24 h. Incubation of cells at 3% O_2_ for 4 days was performed to mimic long-term hypoxia. Conditioned medium was prepared by incubating MDA.MB.231 cells with both shRNA constructs as well as ± Doxycycline for 4 days in hypoxia (3% O_2_). The medium was collected and centrifuged at 1,200 rpm for 5 min to remove cell debris.

For γ-H2A.X staining, cells were treated with 0.5 µM Etoposide (Sigma-Aldrich) for 48 h prior to experiment. Concentration of Etoposide was increased to 1 µM for Western blot of γ-H2A.X/H2A.X. To investigate recovery from DNA damage, cells were starved for 2 days to synchronize cells followed by full medium for 24 h and afterward treated with 250 ng/mL Neocarzinostatin (NCS; Sigma-Aldrich) for 30 min followed by medium change for 4 h recovery.

### 2.3 Orthotopic transplantation, irradiation and Olaparib treatment

A total of 500,000 MDA.MB.231 cells dissolved in 1:1 DPBS (Gibco) and Cultrex^®^ Basement Membrane Extract (R&D) in a total volume of 50 µL were transplanted in the right or in both inguinal mammary glands of i.p. anesthetized [100 mg/kg Ketavet (Bayer) and 5 mg/kg Rompun (Pfizer) in 0.9% NaCl (B.Braun)] immunocompromised mice (BALB/c Rag2^−/−^; γc^−/−^). Tumor growth and general health status of mice were monitored from transplantation until the end of the experiment at least twice a week. Doxycycline (1 mg/mL; Sigma) together with 1% sucrose (Sigma) was applied via the drinking water to induce constant expression of transfected shRNAs in MDA.MB.231 cells. Irradiation with 9 Gy on two consecutive days was performed using the RS2000 from RadSource. Olaparib (25 mg/kg; Selleckchem) representing a low dose (11) was dissolved in 2.5% DMSO (Sigma-Aldrich) and 10% 2-Hydroxylpropyl-β-cyclodextrin (Sigma) in DPBS and applied p.o. 3 h before the first irradiation and 24 h after the last irradiation. The end point of the experiment was reached if one tumor had a tumor volume of 1.5 cm³ or 21 days after start of Doxycycline treatment.

All animal experiments were approved by legal authorities at the regional council Freiburg (G-20/145) and were performed in accordance with the German law for animal welfare. Female mice were randomly assigned for orthotopic transplantation (OT), irradiation and Olaparib treatment within the cohort. The experimenter performing OT, irradiation, Olaparib treatment, and tumor and body score (welfare of mice) measurement during the experiment was not aware which shRNA is expressed in the mice (blinding).

### 2.4 Flow cytometry of tumor cells in lungs of mice

Mice were sacrificed by CO_2_ and lungs were perfused with 0.4 IU/mL heparin (Sintetica) in 0.9% NaCl. The left lung was dissected, cut into small pieces, and washed with DPBS. Tissue was resuspended in 0.25 mg/mL DNAse I Type IV (Sigma), 1 mg/mL Hyaluronidase Type I-S (Sigma), and 6 mg/mL Crude Collagenase Type IV (Sigma) and stirred for 1 h at 37 °C. Cells were transferred via a 40-µm cell strainer and washed again with DPBS. Cells were resuspended in erythrocyte lysis buffer (8.26 g Nh_4_Cl, 1.0 g KHCO_3_, and 0.037 g EDTA in 1 L of ddH_2_O) and incubated for a few minutes at room temperature. Cells were washed again with DPBS and applied to the CytoFLEX S (Beckman Coulter) detecting co-expressed fluorescence of the shRNA-expressing vectors, and analysis was performed using FlowJo (BD Biosciences).

### 2.5 Histology and immunohistochemistry

After removal of the left lung for flow cytometry and right lung for embedding in 4% paraformaldehyde (PFA), tumors of the same mice were isolated, weighed, and embedded in 4% PFA overnight at 4 °C. Tumors and lungs in PFA were applied to an increasing alcohol series (2× 70%, 1× 80%, 2× 95%, and 3× 99% EtOH for 1 h) followed by xylene (2× for 1 h) and paraffin (2× for 2 h). Finally, tissue was embedded in paraffin and 5-µm sections were obtained using a microtome.

For immunohistochemistry staining, paraffin was removed by xylene (4× for 5 min) and a descending alcohol series (3× 99%, 1× 96%, 1× 70%, and 1× 50% EtOH for 5 min) ending with ddH_2_O. Sections were cooked in a pressure cooker in 0.01 M citrate buffer (pH 6) for 20 min and cooled down to room temperature again. Endogenous peroxidase was blocked by 3% hydrogen peroxide in 10% MtOH followed by blocking of unspecific binding sites as described in the VECTASTAIN Elite ABC-HRP Kit (Biozol). Primary antibody targeting Ki67 (ab15580, abcam) or CD31 (ab281583, Abcam) was diluted 1:200, applied to the sections, and incubated overnight at 4 °C in a wet chamber. On the next day, slides were washed with 0.2% Tween-20 in PBS (PBST) and matching secondary antibody from the VECTASTAIN Elite ABC-HRP Kit (Biozol) was applied for 45 min at room temperature (RT). Washing with PBST was followed by the Avidin–Biotin complex from the VECTASTAIN Elite ABC-HRP kit (Biozol) for 45 min at RT. After washing with PBST, sections were incubated with 3,3′-diaminobenzidine (DAB) substrate (Roche) until sufficient brown staining was observed. Counterstaining of nuclei was performed by Meyers hemalum followed by washing in ddH_2_O for 15 min. Slides were mounted with coverslips using Aquatex^®^ (Sigma-Aldrich) and dried at least overnight. Pictures were taken by the BZ-X810 (Keyence) with the appropriate software and analyzed using ImageJ. For CD31-stained sections, at least three representative pictures were captured and analyzed comparing the CD31 signal to the hemalum signal.

### 2.6 TUNEL staining

For TUNEL staining, the ApopTag^®^ Peroxidase in Situ Apoptosis Detection Kit (Sigma-Aldrich) was used according to the manual. In short, paraffin of sections was removed as described above and antigen demasking was performed using 20 µg/mL Proteinase K in PBST. Followed by washing with ddH_2_O twice. Endogenous peroxidase was quenched as described above and sections were washed again with ddH_2_O twice. Equilibration buffer from the kit was applied to the sections followed by incubation with the TdT Enzyme for 1 h at 37 °C in a wet chamber. Reaction was stopped using Stop/Wash buffer for 10 min at RT followed by washing with PBST (3×) and Anti-Digoxigenin Peroxidase Antibody was applied for 30 min at RT. After further washing with PBST (4×), DAB substrate (Roche) was applied for 30 min at RT followed by rinsing sections in ddH_2_O (3×). Counterstaining of nuclei was performed with Meyers hemalum, sections were mounted with Aquatex^®^ as described above, and slides were dried overnight. Pictures were taken using the BZ-X810 (Keyence) with the applicable software and analyzed using ImageJ.

### 2.7 RNA isolation and qRT-PCR

Total RNA Kit (VWR) was used to isolate RNA from MDA.MB.231 cells according to the manual, and concentration was measured using Ezdrop 1000 (Blue-Ray Biotech). RNA (1 μg) was transcribed into cDNA using the iScript™ cDNA Synthesis Kit (Bio-Rad) according to the manual. For qPCR, SYBR™ Select Master Mix (Thermo Fisher Scientific), forward/reverse primers (**Table 2**), and cDNA (1:30) were mixed and cycled in the CFX384 Touch Real-Time PCR Detection System (Bio-Rad). For the analysis, CFX Manager software was used.

**Table 2:** qPCR forward and reverse primer.

Target	Forward 5′ to 3′	Reverse 5′ to 3′
*ACTB*	AGCACTGTGTTGGCGTACAG	CTCTTCCAGCCTTCCTTCCT
*ANGPT1*	ATGGACTGGGAAGGGAACCG	GCATCAAACCACCATCCTCCTG
*CXCL1*	TCTCACAGCCGCCAGACC	CCCATTCTTGAGTGTGGCTATGAC
*DPP8*	GGCCACAAGGATTTACGCAACAAC	AAGGTAGCGACTCCAGCTGATCT
*DPP9*	TGCAGAAGACGGATGAGTCT	GGAATCTCAGAGTAGAGGAG
*EGF*	GCCCTAAGTCGAGACCGGAA	CGGGTGAGGAACAACCGCTA
*EPCAM*	TAAGGCCAAGCAGTGCAACG	TCTCCTTCTGAAGTGCAGTCCG
*FN1*	CCGCCGAATGTAGGACAAGAA	TGCCCACGGTAACAACCTCT
*GAPDH*	CGACCACTTTGTCAAGCTCA	AGGGGTCTACATGGCAACTG
*HIF1a*	GCGCGAACGACAAGAAAAAGATAA	GTGGCAACTGATGAGCAAGC
*KRT5*	AGGGCGAGGAATGCAGACTCA	TGCTACCTCCGGCAAGACCT
*KRT8*	CCTGCAGGAAGGGATCTCCG	GCAGGCTCTGGTTGACCGTA
*PIGF*	TGGAGCACCACTGATAGAGTTGG	GAGATGGGCCATACCTGCCA
*TNC*	GCAGTGCGTGTGCCATGAA	TCTTTGGGAGGAGACACCTCTG
*VEGF*	AGGCCAGCACATAGGAGAGA	TTTCTTGCGCTTTCGTTTTT
*VIM*	CATCGACAAGGTGCGCTTCC	TCTCCTCCTGCAATTTCTCCCG
*ZEB1*	TCCTCTCGAATGAGCACGTG	GCTTGCTTGACTTTCAGCCC

### 2.8 Protein isolation and Western blot

For protein isolation, MDA.MB.231 cells were rinsed in pre-chilled DPBS on ice (3×) and harvested in RIPAplus buffer (50 mM Tris-HCl at pH 7.4; 150 mM NaCl; 1 mM EDTA at pH 7; 2.5 mM Na_4_P_2_O_7_; 1 mM β-glycerophosphate; 1% Triton X-100; 0.001 g/mL SDS; 0.005 g/mL sodium deoxycholate; 1 mM sodium orthovanadate; a PhosSTOP™ tablet/10 mL; and a cOmplete™ ULTRA tablet/10 mL in ddH_2_O) using a cell scraper. Lysates were incubated on ice for 15 min with frequent vortexing and mechanically disrupted by a biovortexer followed by centrifugation at 800 rcf and 4 °C for 15 min. Protein concentration was assessed using the Pierce™ BCA Protein Assay Kit (Thermo Fisher Scientific), and 25 µg of protein was mixed with 5× protein-loading buffer (250 mM Tris-HCl at pH 6.8; 500 mM DTT; 10% SDS; 0.5% bromophenol blue; and 50% glycerol in ddH_2_O) and incubated for 5 min at 95 °C.

For the detection of γ-H2A.X/H2A.X, DPP8, and DPP9, protein lysates were obtained using a different method. Here, MDA.MB.231 cells were also washed in pre-chilled DPBS on ice (3×) but harvested in reducing Laemmli buffer (0.125 M Tris-HCl at pH 6.8; 4% SDS; 20% glycerol; 0.004% bromophenol blue; and 100 mM DTT). Lysates were incubated in liquid nitrogen for a few minutes followed by 95 °C for 10 min.

Prepared lysates were loaded on a gel for SDS-PAGE run at 60 V for 30 min, followed by 120 V for approximately 2 h. Wetblot to transfer proteins on nitrocellulose membranes was performed at 400 mA for approximately 90 min. Generated membranes were blocked in 3% BSA in 0.1% PBST for 1 h and afterward incubated with a primary antibody overnight at 4 °C. Primary antibodies targeting DPP8 (ab42075, Abcam; 1:500), DPP9 (ab42080, Abcam; 1:500), Vimentin (VIM: 550513, BD Pharmingen; 1:500), Keratin 5 (KRT5: PRB-160P, Covance; 1:500), H2A.X (2595, Cell Signaling; 1:500), γ-H2A.X (9718, Cell Signaling; 1:500), TUBA (T9026, Sigma-Aldrich; 1:1000), or GAPDH (97166, Cell Signaling; 1:1,000) were used. After washing with 0.1% PBST for 10 min (3×), membranes were incubated with a secondary antibody for approximately 90 min and rinsed again (3×). As secondary antibodies, IRDye^®^ 680RD Donkey-α-Mouse (LI-COR Biotech; 1:10,000) or IRDye^®^ 800CW Donkey-α-Rabbit (LI-COR Biotech; 1:10,000) was used. Membranes were captured using the Odyssey CLx Imager (LI-COR Biotech) and quantified with Image Studio Lite (LI-COR Biotech).

### 2.9 DPP enzyme activity assay

DPP activity assay was performed as described in (4). Shortly, *shDPP8* or *shDPP9* MDA.MB.231 cells were cultured for 48 h with or without Doxycycline (control) to induce DPP8 or DPP9 deficiency, respectively. Afterwards, cells were detached and incubated in hypotonic buffer (20 mM HEPES, pH 7.9, 1.5 mM MgCl_2_, 10 mM KCl, 1 mM DTT, and 0.5% Triton X-100 in ddH_2_O) on ice for 15 min. Protein concentration was measured using the Pierce™ BCA Protein Assay Kit (Thermo Fisher Scientific). Protein lysate (5 µg) was mixed with H-Gly-Pro-AMC substrate (Bachem), and cleavage of the substrate was measured by increasing AMC signal every minute for 30 min. The slope between 10 and 30 min was calculated and normalized to the control sample.

### 2.10 MTT assay

To assess the viability of HUVECs upon cultivation of with tumor cell conditioned medium, 10,000 HUVECs were seeded in each well of a 96-well plate in EGM-2 medium (Lonza). On the next day, medium of HUVECs was removed and replaced by conditioned medium of MDA.MB.231 cells. This was incubated for 48 h at 37 °C and 5% CO_2_ followed by a medium change to indicator-free DMEM (Pan Biotech) mixed 1:10 with 5 mg/mL Thiazolyl Blue Tetrazolium Blue (MTT; Sigma-Aldrich) and incubated for 2 h. Afterwards, MTT-containing medium was replaced by DMSO (Sigma-Aldrich) for 20 min and measured at 570 nm in the CLARIOstar plate reader (BMG Labtech).

### 2.11 Transwell assay

To assess the migratory capacity of HUVECs toward tumor cell conditioned medium, conditioned medium of MDA.MB.231 cells was transferred in the lower chamber of the Transwell plate, whereas 80,000 HUVECs were seeded in the upper chamber of the Transwell plate in serum-free medium. This was incubated for 48 h at 37 °C and 5% CO_2_. HUVECs on the upper part of the Transwell were removed by a swab followed by staining with crystal violet. Migrated HUVECs were imaged using the BZ-X810 (Keyence) with the appropriate software and eight pictures/Transwell were analyzed using ImageJ.

### 2.12 Scratch assay

Scratch assay was performed as described in (4). Shortly, MDA.MB.231 cells were incubated until 100% confluence, and a scratch was introduced using a pipette tip. Pictures were taken every hour for 48 h by the JuLI™ Stage (NanoEntek) and analyzed using the JuLI™ Edit software (NanoEntek).

### 2.13 γ-H2A.X staining

Tumor cells were treated with either Etoposide or NCS as described in Section 2.2, harvested, and fixed with 4% PFA for 10 min at RT. After rinsing cells in DPBS, cells were permeabilized by 0.1% Triton X-100 in DPBS for 5 min at RT followed by washing cells in DPBS. APC/Fire™ 750 anti-γ-H2A.X (BioLegend; 1:100) was incubated on cells for 10 min at RT in the dark and again washed with DPBS. Cells were applied to the CytoFLEX S (Beckman Coulter) to detect fluorescent cells, and analysis was performed using FlowJo (BD Biosciences).

### 2.14 Data analysis, presentation, and statistics

Data in line or bar graphs were expressed as mean ± SEM or mean + SEM, respectively. *n* represents independent biological experiments. For OT, *n* represents different mice, whereas cohorts of OTs are labeled in different colors in the graph. Statistical analyses were done with Prism 10 (GraphPad), comparison of two groups was done by two-sided two- or paired-sample *t*-test, and comparison of multiple groups was done by one-way analysis of variance (ANOVA) with Šídák test.

## 3 Results

### 3.1 DPP9 deficiency increased tumor weight and metastasis formation

**Figure 1:**
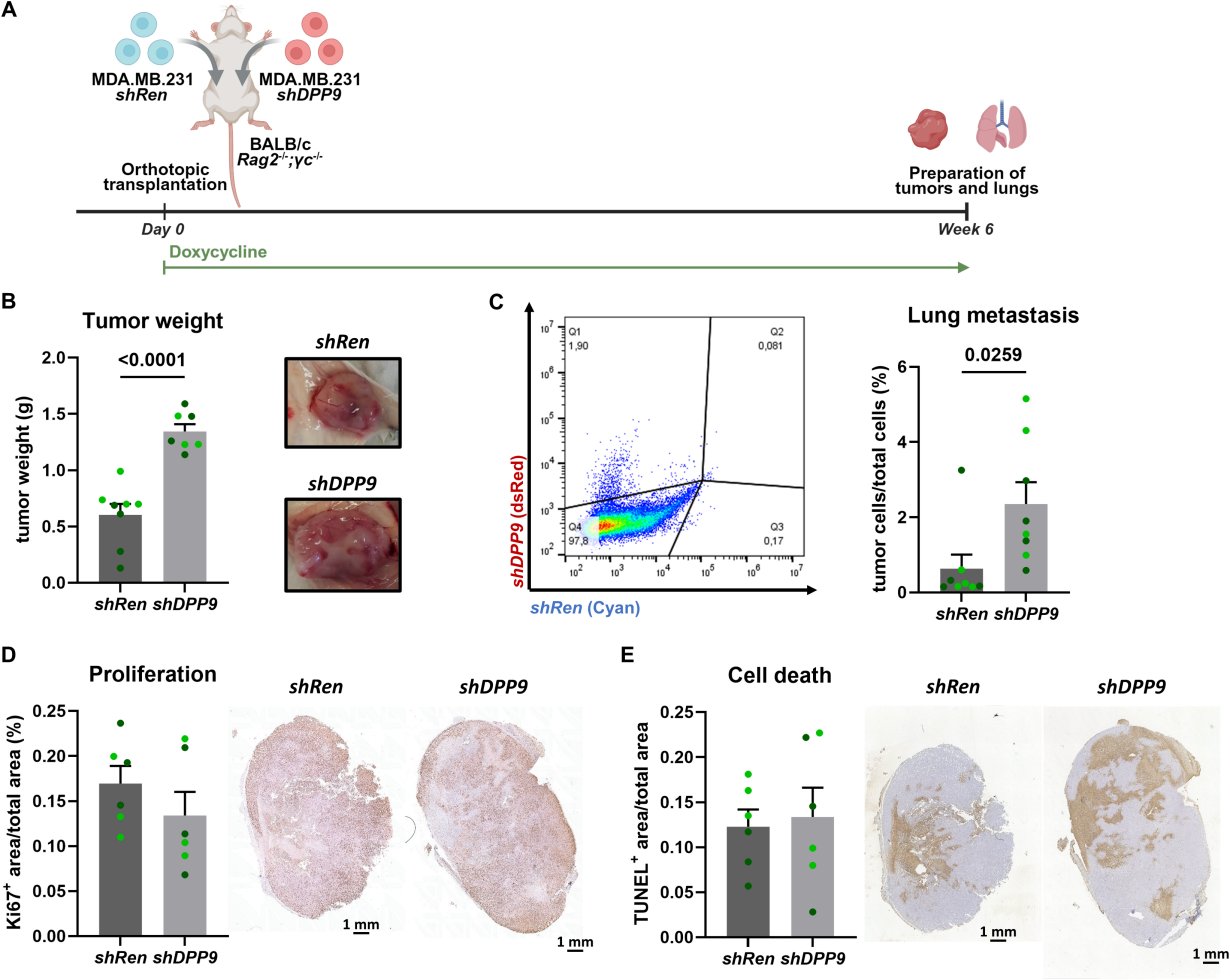
DPP9 deficiency increased tumor weight and metastasis formation. **(A)** Schematic description of orthotopic transplantation procedure. Transplantation of *shRen* (control: Blue) and *shDPP9* (red) MDA.MB.231 cells in either the right or the left mammary fat pad of immunocompromised mice, respectively. Mice were treated with Doxycycline during the whole experiment ending after 6 weeks. (Created in BioRender). **(B, C)** Tumor weight **(B)** and lung metastasis measured by FACS **(C)** analyzed at the end point of the experiment [*shRen*: *n* = 8 mice; *shDPP9*: *n* = 7 mice **(B)** or *n* = 8 mice **(C)** in two independent experiments]. **(D, E)** Ki67 staining indicating proliferation **(D)** and TUNEL staining indicating cell death **(E)** of end point tumors (*n* = 6 mice in two independent experiments). Bar graphs show mean + SEM and *p*-value is calculated by two-sample *t*-test.

To investigate the impact of DPP9 on tumor growth and metastasis formation, MDA.MB.231 cells expressing either a non-targeting shRNA (*shRen*) used as control or DPP9-targeting shRNA (*shDPP9*) were orthotopically transplanted in the same immunodeficient mice in either the right or the left mammary gland (**Figure 1A**). Mice received Doxycycline via the drinking water from time point of OT until the end of the experiment 6 weeks after OT. Induction of shRNA expression upon Doxycycline was verified *in vitro* by flow cytometry of co-expressed fluorescence for *shRen* (**Supplementary Figure 1A**) and *shDPP9* (**Supplementary Figure 1B**). Successful targeting of DPP9 by the shRNA was further validated on mRNA level (**Supplementary Figure 1C**), protein level (**Supplementary Figure 1D**), and enzyme activity (**Supplementary Figure 1E**). Interestingly, DPP9 enzyme activity was higher than DPP8 activity in MDA.MB.231 cells. Weight of tumors with DPP9 deficiency was significantly increased compared to control tumors (**Figure 1B**). Furthermore, significantly more DPP9-deficient MDA.MB.231 cells than control tumor cells were measured in lungs of mice (**Figure 1C**). Histological analysis of Ki67, a marker for proliferation, did not show any difference in the stained area of end point tumors between both genotypes (**Figure 1D**). Furthermore, TUNEL staining, representing cell death, demonstrated no difference in stained area as well (**Figure 1E**). These results show a strong tumor growth as well as metastasis formation upon DPP9 deficiency in a TNBC cell line and are in line with the clinical association of low DPP9 and poor prognosis (4).

### 3.2 DPP9 deficiency promoted vessel formation in early tumor development

**Figure 2:**
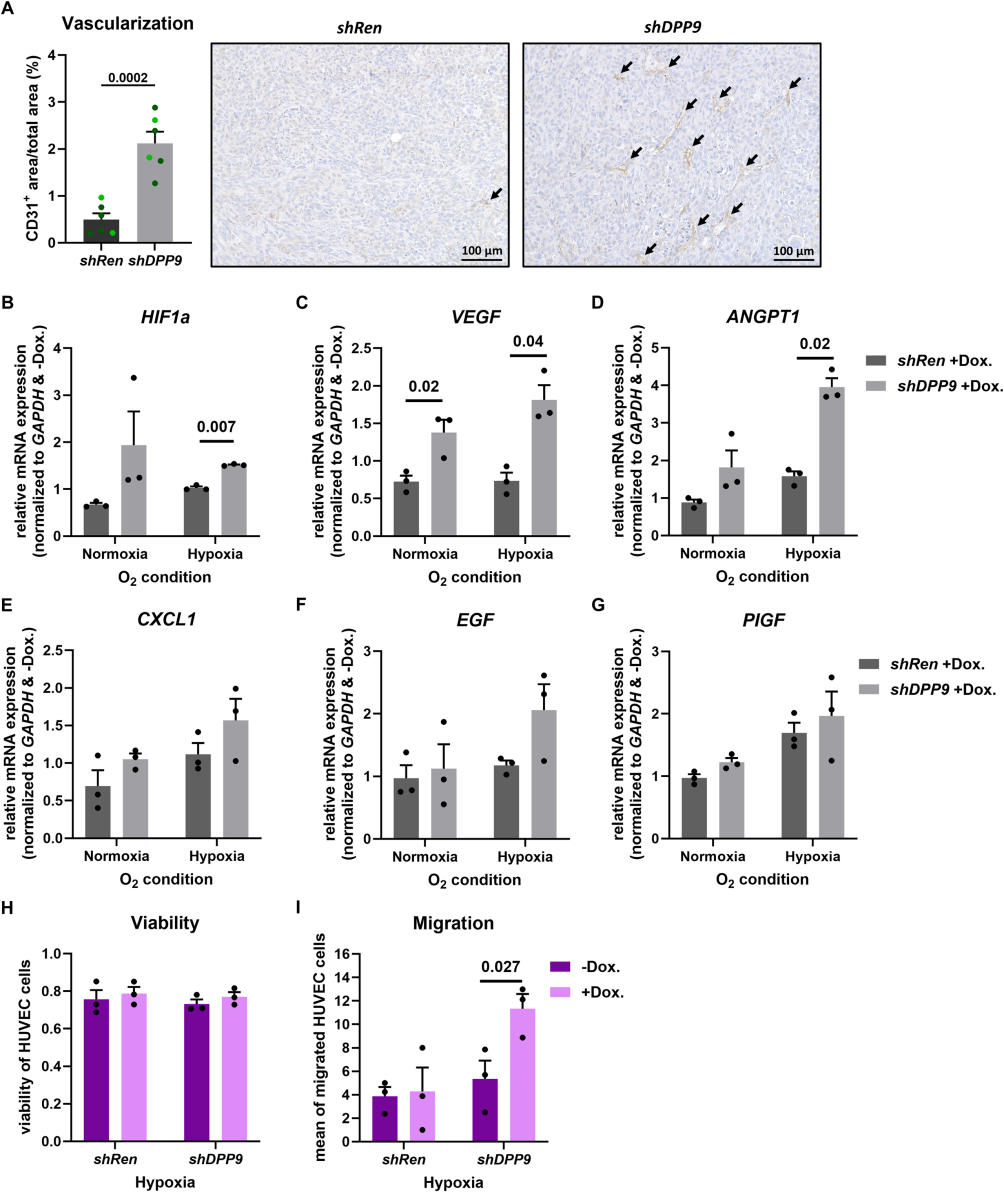
DPP9 deficiency promoted vessel formation in early tumor development. **(A)** CD31 staining indicating endothelial cells of end point tumors (*n* = 6 mice in two independent experiments). **(B–G)** mRNA expression of *HIF1a* **(B)**, *VEGF* **(C)**, *ANGPT1* **(D)**, *CXCL1* **(E)**, *EGF* **(F)**, and *PIGF* **(G)** in *shRen* or *shDPP9* MDA.MB.231 cells ± Doxycycline in normoxia (21% O_2_) or hypoxia (3% O_2_) (*n* = 3 independent biological replicates). **(H)** MTT assay of HUVECs cultured in hypoxic tumor cell conditioned medium for 48 h (*n* = 3 independent biological replicates of HUVECs and tumor cell conditioned medium). **(I)** Transwell assay of HUVECs cultured in serum-free medium migrating toward hypoxic tumor cell conditioned medium for 48 h (*n* = 3 independent biological replicates of HUVECs and tumor cell conditioned medium). Bar graphs show mean + SEM and *p*-value is calculated by paired-sample *t*-test.

Neither proliferation nor cell death was affected by DPP9 deficiency at the end point of tumor development. Therefore, vessel formation in tumor sections was assessed by staining for CD31, a marker expressed in endothelial cells. Interestingly, DPP9-deficient breast tumors had significantly more CD31-positive cells compared to control tumors (**Figure 2A**). Vessel formation in tumors is mostly initiated by hypoxic conditions during early tumorigenesis upon uncontrolled proliferation of tumor cells (12). Thus, *shRen* and *shDPP9* MDA.MB.231 cells with or without Doxycycline were cultured *in vitro* in normoxia (21% O_2_) as well as hypoxia (3% O_2_) and mRNA expression of angiogenesis-inducing proteins was measured. *HIF1a* (**Figure 2B**), *VEGF* (**Figure 2C**) and *ANGPT1* (**Figure 2D**) show already in normoxia tendencies to higher mRNA expression upon DPP9 deficiency, . This increase in mRNA levels gets significant for the afore described markers incubating cells in hypoxic conditions. A similar tendency to higher mRNA expression in DPP9-deficient MDA.MB.231 cells upon hypoxia was also demonstrated for *CXCL1* (**Figure 2E**), *EGF* (**Figure 2F**), and *PIGF* (**Figure 2G**), although the difference is not significant. The effect of release of such angiogenic factors into the medium of MDA.MB.231 cells upon hypoxia was measured assessing viability (**Figure 2H**) and migratory capacity (**Figure 2I**) of endothelial HUVECs *in vitro* using hypoxic tumor cell conditioned medium (4 days). Tumor conditioned medium had no impact on the viability of HUVECs independent of DPP9 deficiency. However, more HUVECs migrated toward hypoxic tumor cell conditioned medium of DPP9-deficient MDA.MB.231 cells compared to all control conditions. Therefore, faster tumor growth and metastases might be supported by enhanced endothelial cell migration promoting vessel formation upon DPP9 deficiency in early tumor development (13).

### 3.3 DPP9 deficiency had no impact on mesenchymal phenotype of MDA.MB.231 cells

**Figure 3:**
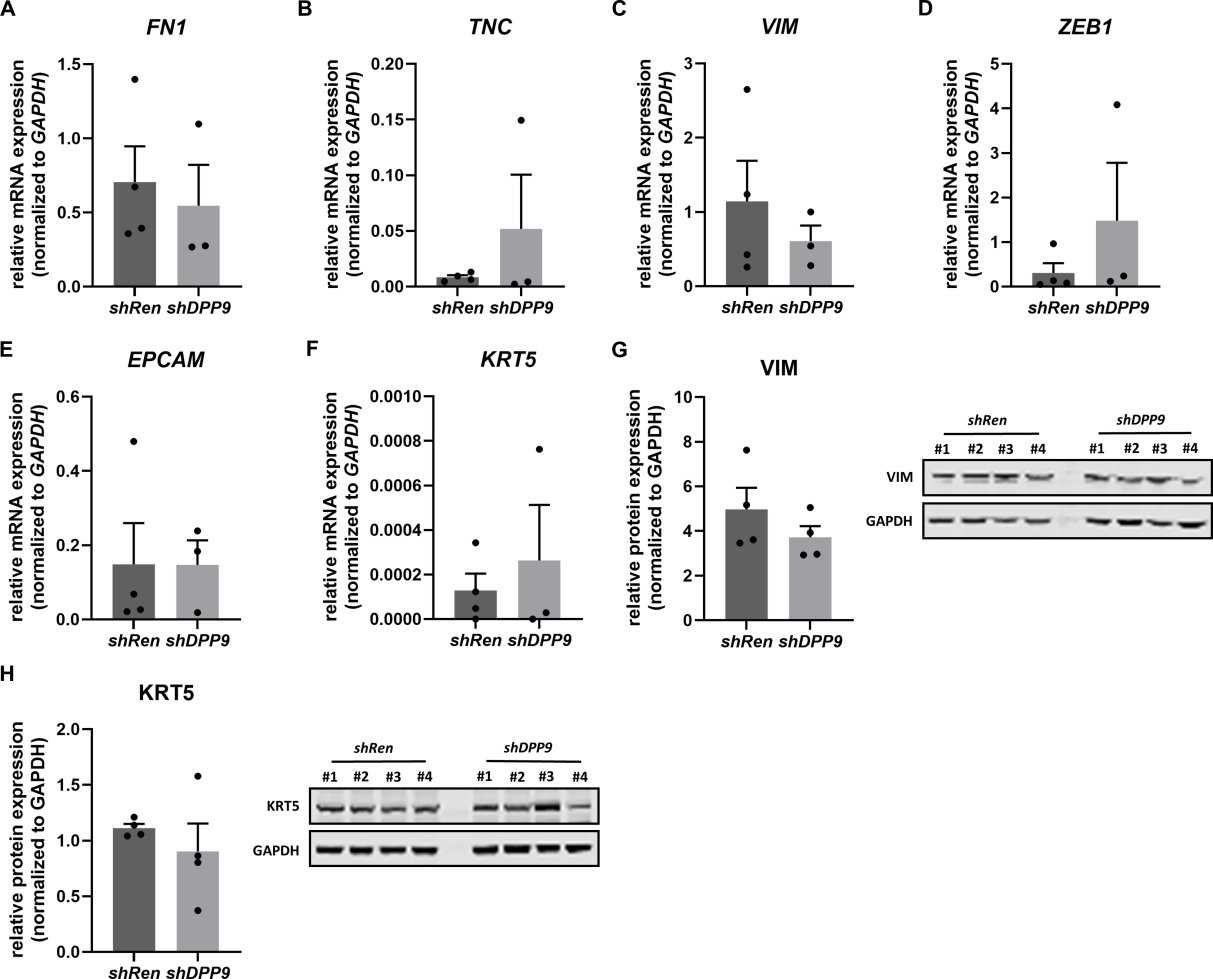
DPP9 deficiency had no impact on mesenchymal phenotype of MDA.MB.231 cells *in vivo*. **(A–F)** mRNA expression of *FN1* **(A)**, *TNC* **(B)**, *VIM* **(C)**, *ZEB1* **(D)**, *EPCAM* **(E)**, and *KRT5* **(F)** in end point tumors (*n* = 4 mice). **(G, H)** Protein expression of VIM **(G)** and KRT5 **(H)** in end point tumors (*n* = 4 mice). Bar graphs show mean + SEM and *p*-value is calculated by two-sample *t*-test.

In general, increased vessel formation contributes to stronger metastasis formation. However, a more migratory and invasive behavior triggered by a more mesenchymal phenotype of cells increases the capability of cells to form metastasis in distant organs (14). Therefore, mRNA expression of various epithelial as well as mesenchymal markers was measured in end point tumors. Because of the already mesenchymal phenotype of MDA.MB.231 cells, high mRNA expression of *FN1* (**Figure 3A**), *TNC* (**Figure 3B**), *VIM* (**Figure 3C**), and *ZEB1* (**Figure 3D**) was measured for both genotypes. *TNC* and *ZEB1* demonstrated a tendency to higher mRNA expression upon DPP9 deficiency, whereas *FN1* and *VIM* had a propensity to reduced mRNA expression upon DPP9 deficiency. No strong difference in the mRNA expression of epithelial markers like *EPCAM* (**Figure 3E**) and *KRT5* (**Figure 3F**) was measured. On the protein level, similar expression of VIM (**Figure 3G**) as well as KRT5 (**Figure 3H**) was analyzed in MDA.MB.231-derived tumors independent of the genotype. Analysis of MDA.MB.231 cells *in vitro* also did not show any changes in mRNA expression of *FN1* (**Supplementary Figure 2A**), *TNC* (**Supplementary Figure 2B**), *VIM* (**Supplementary Figure 2C**), *ZEB1* (**Supplementary Figure 2D**), *EPCAM* (**Supplementary Figure 2E**), and *KRT8* (**Supplementary Figure 2F**), another epithelial marker. As a functional readout, migratory behavior of the cells was analyzed *in vitro* by measuring the ability of *shRen* and *shDPP9* MDA.MB.231 cells with or without Doxycycline to migrate into an introduced gap (**Supplementary Figure 2G**). MDA.MB.231 cells closed the gap after approximately 40 h independent of the genotype and had no further advantage by adding TGF-β, which usually promotes migration of cells. Owing to similar expression of well-established epithelial and mesenchymal markers in MDA.MB.231-derived tumors as well as MDA.MB.231 cell culture, no change in their migratory behavior was expected. Therefore, increased metastasis formation upon DPP9 deficiency seems to be mainly mediated by the demonstrated enhanced vessel formation increasing the ability of cells to reach the bloodstream and thereby distant organs.

### 3.4 DPP9 deficiency reduced efficiency of DNA damage repair

**Figure 4:**
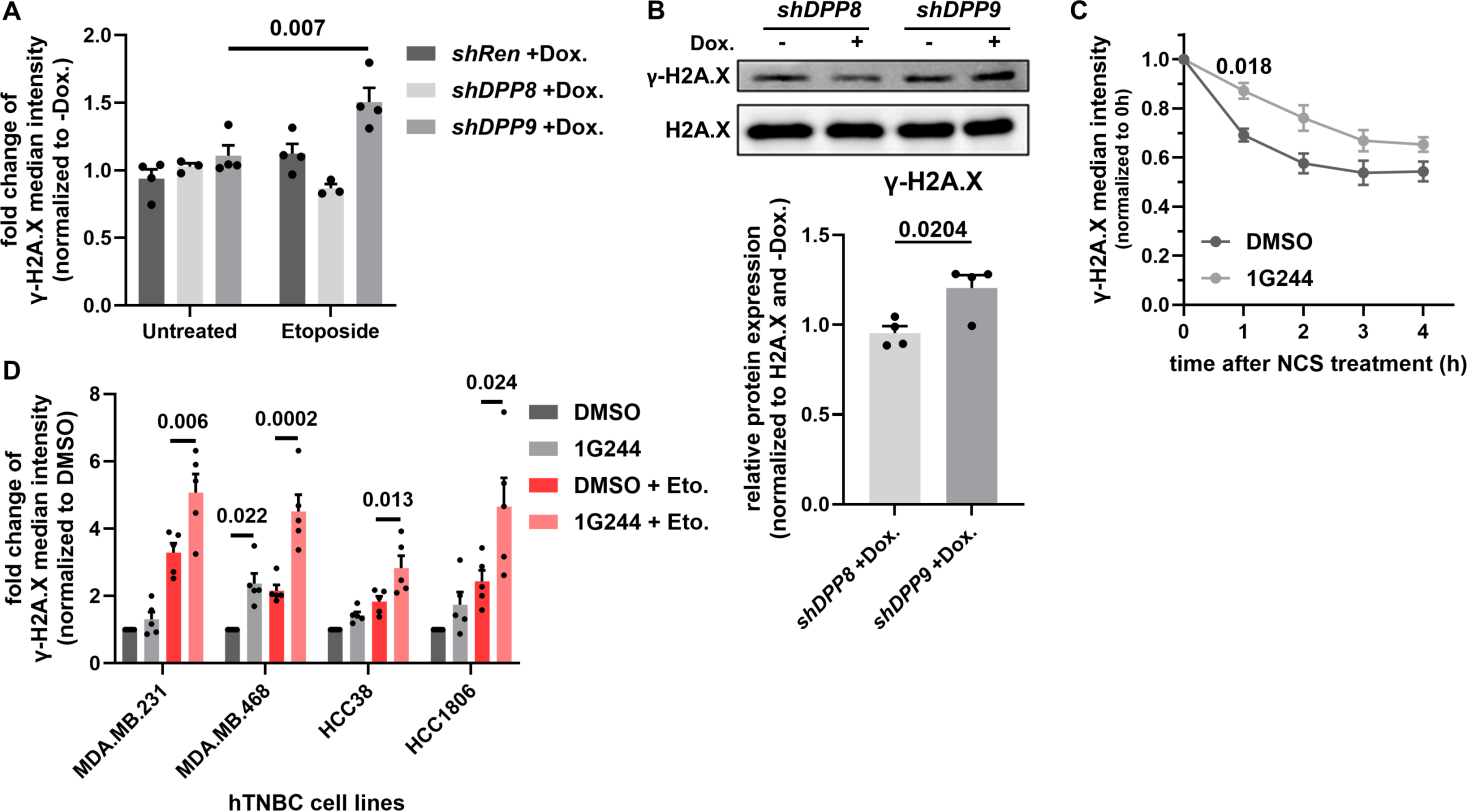
DPP9 deficiency reduced efficiency of DNA damage repair initiation. **(A, B)** Measurement of γ-H2A.X by flow cytometry **(A)** and Western blot **(B)** of *shRen*, *shDPP8*, or *shDPP9* MDA.MB.231 cells ± Doxycycline and ± 0.5 µM Etoposide **(A)** or 1 µM Etoposide **(B)** for 48 h, respectively (*n* = 4 independent biological replicates). **(C)** Measurement of γ-H2A.X by flow cytometry of MDA.MB.231 cells ± 1G244 and ± 250 ng/mL Neocarzinostatin (NCS) for 30 min (*n* = 4 independent biological replicates). **(D)** Measurement of γ-H2A.X by flow cytometry of MDA.MB.231, MDA.MB.468, HCC38, and HCC1806 cells ± 1G244 and ± 0.5 µM Etoposide for 48 h (*n* = 5 independent biological replicates). Bar and line graphs show mean + SEM and ± SEM, respectively, and *p*-value is calculated by one-way ANOVA with Šídák test **(A, D)** or paired-sample *t*-test **(B, C)**.

It is known that hypoxia is inducing DNA damage and DPP9 is described to have a role in DNA damage response. DPP9 was shown to degrade BRCA2 upon double-strand break (DSB) induction to enable efficient HR (10). Consequently, reduced expression of DPP9 may result in an HR defect as it is already described for BRCA1/2 deficiency (15), which might contribute to the increased tumor growth upon DPP9 deficiency. To test whether DPP9 deficiency in MDA.MB231 cells increase DNA damage, *shDPP9* MDA.MB.231 cells with Doxycycline (DPP9 deficiency) or without Doxycycline (control) were treated with Etoposide to induce DNA damage, and phosphorylation of histone H2A.X (γ-H2A.X) was visualized either by flow cytometry (**Figure 4A**) or Western blot (**Figure 4B**). As expected, DPP9-deficient MDA.MB.231 cells showed more γ-H2A.X in both approaches compared to control cells. Interestingly, no increase in γ-H2A.X was measured upon deficiency of DPP8 (*shDPP8*), verifying this unique function of DPP9 already described (10). DPP8 deficiency was validated on the mRNA level (**Supplementary Figure 3A**) and protein level (**Supplementary Figure 3B**) and by reduced enzyme activity (**Supplementary Figure 3C**). Furthermore, recovery from NCS inducing also DNA damage was slower in MDA.MB.231 cells treated with a DPP8/9 inhibitor (1G244) compared to controls (**Figure 4C**). To show that DPP9 activity is promoting this effect not only in MDA.MB.231 but also in other hTNBC cell lines, MDA.MB.468, HCC38, and HCC1806 cells were treated with 1G244 or solvent control as well as ± Etoposide to induce DNA damage, and γ-H2A.X was measured (**Figure 4D**). All 1G244-treated hTNBC cell lines showed an increase of γ-H2A.X median intensity upon Etoposide compared to solvent controls. Interestingly, MDA.MB.468 and HCC1806 demonstrated a an increase of γ-H2A.X median intensity similar to that of MDA.MB.231 cells. Therefore, low DPP9 levels seem to reduce the efficient repair of induced DNA damage.

### 3.5 DPP9-deficient tumors were less sensitive to irradiation than controls

**Figure 5:**
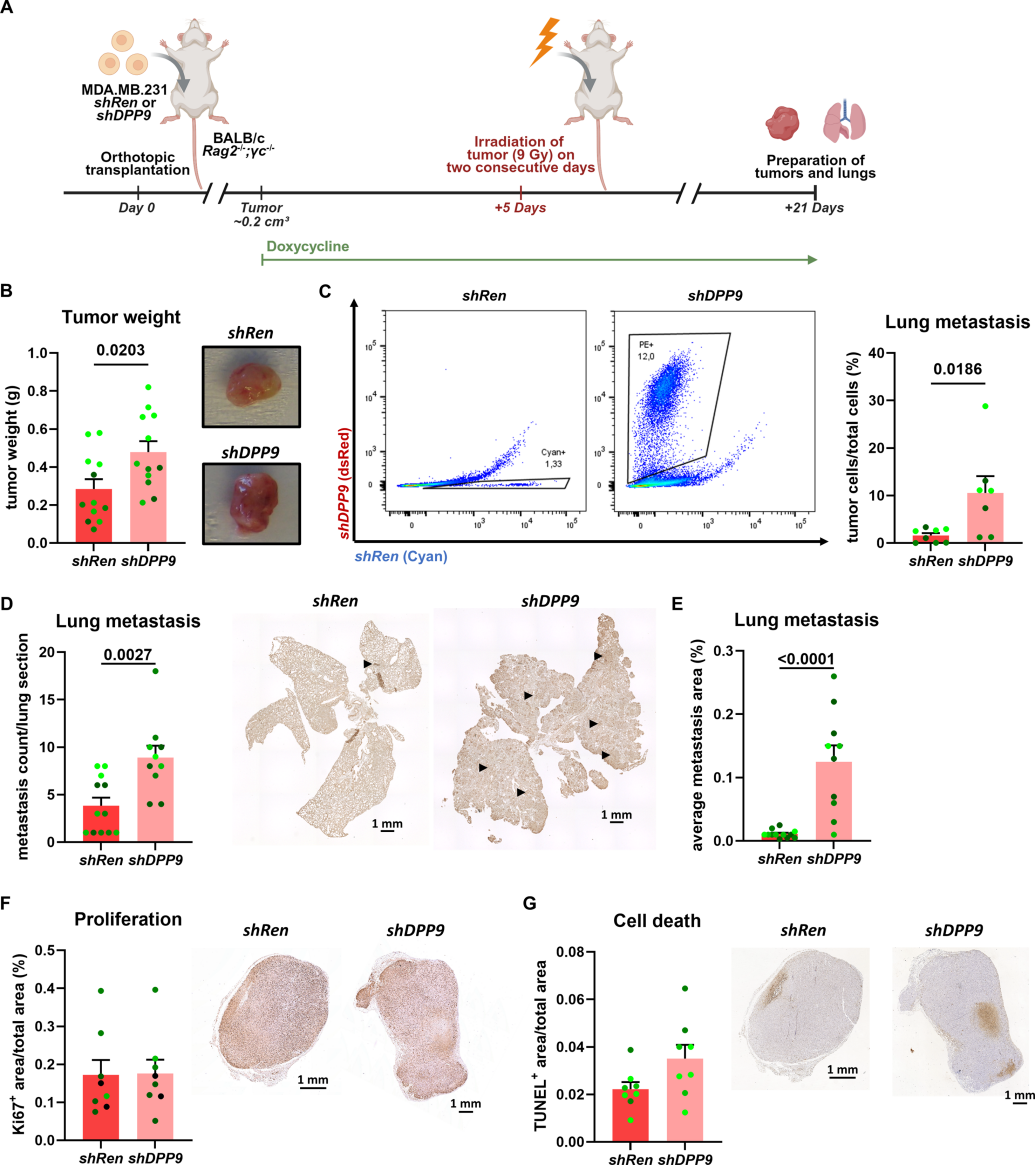
DPP9-deficient tumors were less sensitive to irradiation than controls. **(A)** Schematic description of orthotopic transplantation and irradiation procedure. Transplantation of *shRen* (control) or *shDPP9* MDA.MB.231 cells in the right mammary fat pad of immunocompromised mice. All mice were treated with Doxycycline when tumors reached a size of approximately 0.2 cm³. Five days after the start of Doxycycline treatment, all mice were locally irradiated with 9 Gy on two consecutive days. All mice were sacrificed 21 days after the start of Doxycycline treatment. (Created in BioRender). **(B, C)** Tumor weight **(B)** and lung metastasis measured by FACS **(C)** analyzed at the end point of the experiment [*shRen*: *n* = 12 mice **(B)** or *n* = 8 mice **(C)**; *shDPP9*: *n* = 12 mice **(B)** or *n* = 7 mice **(C)** in 3 independent experiments]. **(D, E)** Metastasis count **(D)** and average metastasis area **(E)** of Ki67-stained lungs (*shRen*: *n* = 12 mice; *shDPP9*: *n* = 10 mice in 3 independent experiments). **(F, G)** Ki67 staining indicating proliferation **(F)** and TUNEL staining indicating cell death **(G)** of end point tumors (*n* = 8 mice in three independent experiments). Bar graphs show mean + SEM and *p*-value is calculated by two-sample *t*-test.

Irradiation inducing unspecific DNA damage represents a standard therapy for TNBC (3). Because of the shown influence of DPP9 on DNA damage repair, irradiation might be a valuable treatment option for patients with breast cancer with low levels of DPP9, because cells are not able to repair the irradiation-induced DNA damage efficiently. To test this hypothesis, mice were orthotopically transplanted with either MDA.MB.231 with DPP9 deficiency (*shDPP9*) or control MDA.MB.231 cells (*shRen*) (**Figure 5A**). Doxycycline treatment started at a tumor size of approximately 0.2 cm³ to have similar sized tumors at the start of the therapy (**Supplementary Figure 4A**). Five days after the start of shRNA induction, local irradiation of all tumors was performed on two consecutive days with 9 Gy each. Preparation of mice was done 21 days after start of Doxycycline treatment. Although all tumors independent of shRNA-mediated deficiency responded to irradiation with an initial reduction of tumor size (**Supplementary Figure 4B**), irradiated DPP9-deficient tumors started to grow faster and had at the end of the experiment a significantly higher tumor weight compared to irradiated control tumors (**Figure 5B**). Additionally, significantly more tumor cells were measured in lungs of mice due to DPP9 deficiency (**Figure 5C**). Furthermore, a higher metastasis count (**Figure 5D**) as well as average metastasis area (**Figure 5E**) were measured in Ki67-stained lungs of mice with DPP9-deficient tumors. Neither staining of tumor sections with Ki67 (**Figure 5F**) nor TUNEL staining (**Figure 5G**) demonstrated any difference in the stained area comparing both genotypes. These results demonstrate that irradiation leads to higher tumor weight as well as more metastasis formation upon low levels of DPP9.

### 3.6 DPP9 deficiency sensitized for combinatory treatment with irradiation and Olaparib

**Figure 6:**
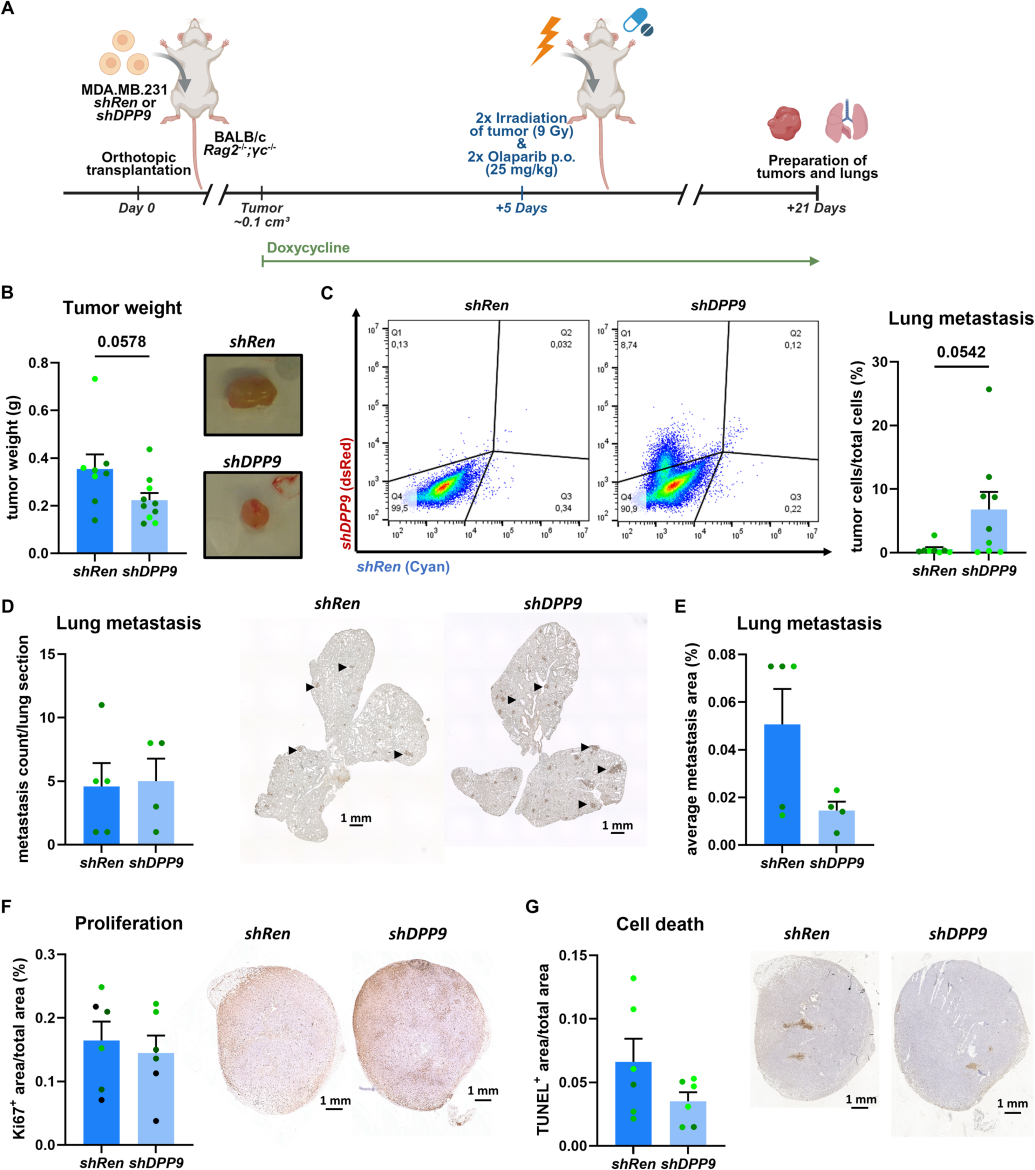
DPP9 deficiency sensitized for combinatory treatment with irradiation and Olaparib. **(A)** Schematic description of orthotopic transplantation and combinatory treatment procedure. Transplantation of *shRen* (control) or *shDPP9* MDA.MB.231 cells in the right mammary fat pad of immunocompromised mice. All mice were treated with Doxycycline when tumors reached a size of approximately 0.1 cm³. Five days after start of Doxycycline treatment, all mice were locally irradiated with 9 Gy on two consecutive days. Irradiation of mice on the first day was preceded by 25 mg/kg Olaparib p.o. and Olaparib treatment was repeated once again 24 h after last irradiation. All mice were sacrificed 21 days after start of Doxycycline treatment. (Created in BioRender). **(B, C)** Tumor weight **(B)** and lung metastasis measured by FACS **(C)** analyzed at the end point of the experiment [*shRen*: *n* = 8 mice **(B)**; *shDPP9*: *n* = 10 mice **(B)** or *n* = 9 mice **(C)** in four independent experiments]. **(D, E)** Metastasis count **(D)** and average metastasis area **(E)** of Ki67-stained lungs (*shRen*: *n* = 5 mice; *shDPP9*: *n* = 4 mice in three independent experiments). **(F, G)** Ki67 staining indicating proliferation **(F)** and TUNEL staining indicating cell death **(G)** of end point tumors (*n* = 6 mice in four independent experiments). Bar graphs show mean + SEM and *p*-value is calculated by two-sample *t*-test.

As shown above, low DPP9 levels impaired the repair of induced DNA damage as it is already known for BRCA1/2 deficiencies. Owing to the common use of PARP inhibitors like Olaparib in BRCA1/2-deficient patients with breast cancer, we tested in our mouse model if irradiation in combination with Olaparib results in synthetic lethality upon DPP9 deficiency. For this purpose, mice were treated with 25 mg/kg Olaparib p.o. 3 h before the first irradiation and 24 h after the last irradiation (**Figure 6A**). Start of treatment was at a tumor size of approximately 0.1 cm³ (**Supplementary Figure 4C**). Here, all tumors responded to the combinatory treatment initially with slow tumor growth getting exponential about a week after treatment (**Supplementary Figure 4D**). Nevertheless, control tumors (*shRen*) grew out faster than DPP9-deficient tumors upon combinatory treatment (**Figure 6B**). Despite this result, DPP9-deficient tumors showed a high variation in metastasis formation but tended to generate more metastasis in the lungs of mice compared to controls (**Figure 6C**). Interestingly, metastasis count (**Figure 6D**) was slightly increased, whereas average metastasis area (**Figure 6E**) was decreased in Ki67-stained lungs of mice with DPP9-deficient tumors, underscoring the mixed outcome concerning metastasis formation. Again, Ki67 (**Figure 6F**) and TUNEL staining (**Figure 6G**) did not show any difference in the stained area of DPP9-deficient tumors compared to controls. These results show the effectiveness of combinatory treatment with irradiation/PARP inhibition of mice with DPP9-deficient triple-negative breast tumors.

## 4 Discussion

**Figure 7:**
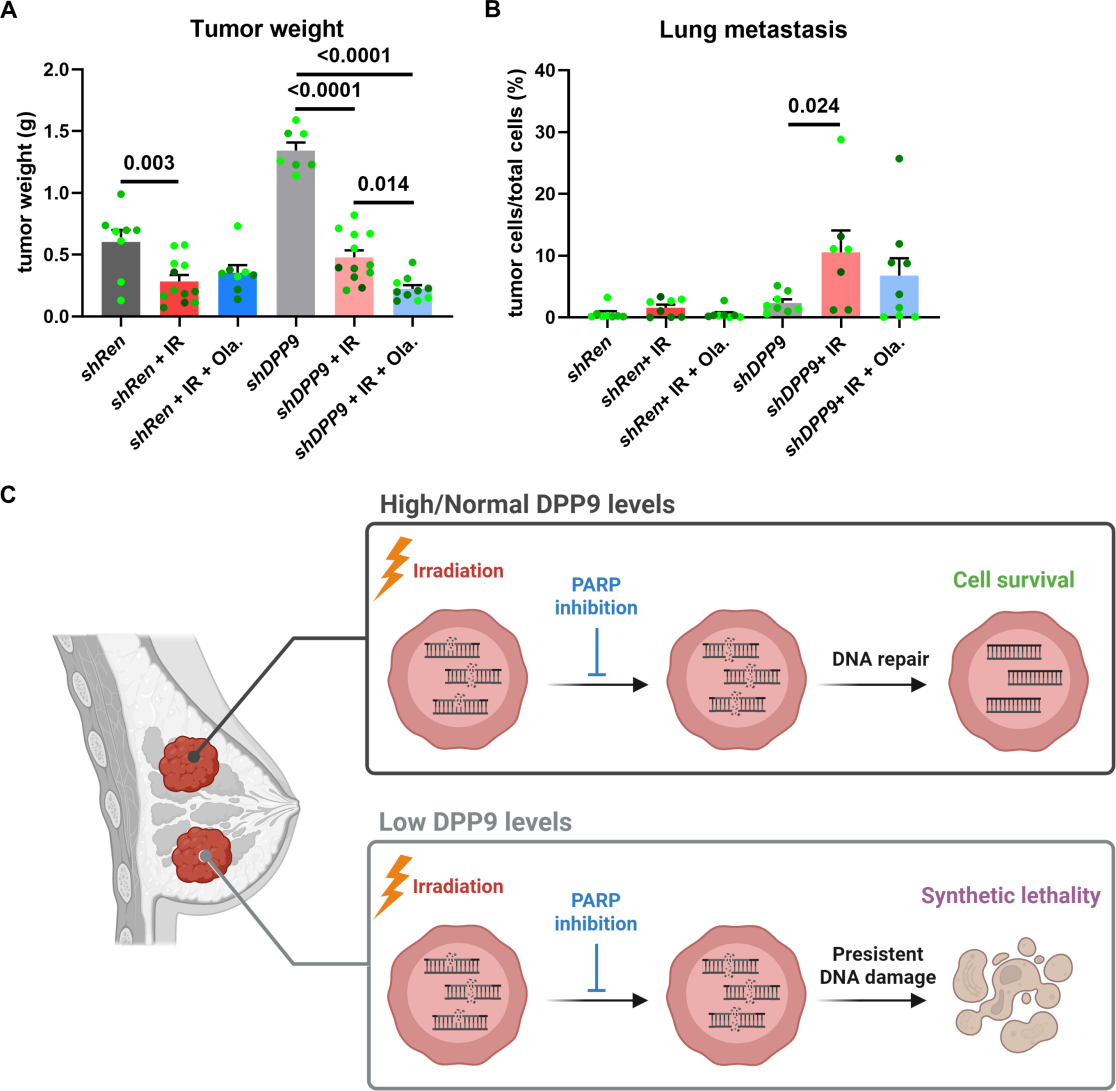
Summary of the effect of low DPP9 levels, local irradiation, or combinatory treatment. **(A, B)** Tumor weight **(A)** and lung metastasis measured by FACS **(B)** analyzed at the end point of the experiments [tumor weight **(A)** is data from **Figure 1B**, **5B**, **6B** and lung metastasis **(B)** is data from **Figure 1C**, **5C**, **6C**]. Bar graphs show mean + SEM and *p*-value is calculated by one-way ANOVA with Šídák test. **(C)** Summary of the results showing that low DPP9 levels sensitizes triple-negative breast cancer cells for combinatory treatment with local irradiation and PARP inhibition (Olaparib). (Created in BioRender).

Here, we clearly demonstrated that combinatory treatment of mice with local irradiation and PARP inhibition using Olaparib is highly effective in reducing tumor growth of already established tumors, but only if tumor cells present with low levels of DPP9 (**Figure 7A**). DPP9 deficiency in MDA.MB.231 cells led to a strong increase in tumor growth compared to controls, showing the impact of DPP9 on the aggressiveness of breast tumors. Non-targeted treatment of triple-negative breast tumors with local irradiation reduced tumor growth strongly independent of the genotype. However, additional Olaparib treatment reduced tumor weight only in mice with DPP9-deficient tumors and did not change tumor weight in control tumors with irradiation. This clearly demonstrated the potential of combined therapy with irradiation and Olaparib in patients with low DPP9 levels, leading to synthetic lethality in these tumor cells. Interestingly, a clinical trial called RADIOPARP combines irradiation with PARP inhibition in patients with TNBC by starting with a daily dose of PARP inhibitor followed by an initial irradiation to improve outcome for patients (16).

In general, HR-deficient tumors independent of their origin are sensitive for irradiation, because it induces DNA damage either directly or indirectly, via generating reactive oxygen species (ROS), which in turn increases genetic instability (17). If DNA damage is too strong, cells will have a lot of misrepair, leading to mitotic catastrophe and subsequent cell death (18, 19). If HR is defective, other mechanisms like non-homologous end-joining (NHEJ) or alternative end-joining (Alt-EJ) will take over to repair DSBs (20). These mechanisms are more error-prone, making misrepair more likely to occur. As a result, most of the cells die. However, some of the HR-deficient cancer cells generate pro-tumorigenic mutations by misrepair and, thus, escape from cell death driving tumor growth (21). A breakthrough in treating HR-deficient tumors was the use of PARP inhibitors (22). PARP1 is an enzyme important not only in the repair of DNA single-strand breaks by single-strand break repair (SSBR) but also in the repair of DSBs by alternative end-joining (Alt-EJ). PARP inhibitors trap PARP1 at the DNA damage site, thereby producing further DNA damage, which requires HR for repair (17). Thus, HR-deficient tumors are more sensitive to combinatory treatment of irradiation and PARP inhibition (23, 24) similar to MDA.MB.231-derived DPP9-deficient tumors in our study compared to HR-proficient tumors (**Figure 7A**).

This phenotype seems to be mainly mediated by the role of DPP9 in DNA damage repair (10). Here, DPP9 was shown to degrade BRCA2 to support HR upon induction of DSBs in the DNA. We verified a defect in DNA damage repair by elevated γ-H2A.X levels upon Etoposide as well as slower DNA damage repair upon NCS in our DPP9-deficient MDA.MB.231 cells. Furthermore, DPP8/9 inhibition in combination with Etoposide treatment in other TNBC cell lines also showed an increase of γ-H2A.X levels compared to controls. Therefore, low DPP9 levels seem to result in defective DNA damage repair. Because the success of PARP inhibition in reducing tumor weight in our mouse model with DPP9-deficient tumors so far has only been shown for HR-deficient tumors (25), we hypothesize that HR is mainly affected by the DPP9 deficiency as described by Bolgi et al. (2022) (10).

Furthermore, stronger vascularization was measured in DPP9-deficient tumors compared to controls, indicating a role of DPP9 in suppressing tumor-driven angiogenesis. In line with this, an upregulation of HIF1-responsive genes known to drive proliferation, migration, and vessel formation of endothelial cells (12) was measured in DPP9-deficient MDA.MB.231 cells. This was even potentiated using hypoxic (3% O_2_) culture conditions demonstrating the importance of DPP9 in reducing angiogenesis, especially upon hypoxia. This might have a strong impact especially during early tumor development (12). Tumor vascularization is already known to promote tumor growth by supplying nutrients as well as to support metastasis formation by increasing the chance for cells to enter the bloodstream and reach distant organs (13). Thus, low levels of DPP9 in triple-negative breast tumors might contribute via increased vessel formation to its aggressive phenotype shown by our mouse model.

Interestingly, hypoxia influences DNA damage repair as well. Under hypoxic conditions, various cancer cell types demonstrated a reduced expression of proteins associated with DNA damage repair (26–28). In particular, HR was affected by this, resulting in less error-free DNA damage repair and, in consequence, an increased genetic instability of cells. Therefore, DPP9 deficiency, especially in combination with irradiation, might potentiate this effect of hypoxia-induced defective DNA damage repair, resulting in the measured increased tumor weight and higher metastatic potential.

Nevertheless, irradiation as well as combinatory treatment with irradiation/PARP inhibition resulted in an increase of tumor cells detected in lungs of mice with DPP9-deficient tumors compared to mice with untreated DPP9-deficient tumors (**Figure 7B**). However, DPP9-deficient tumor cells were not detected in all lungs of mice treated with irradiation or irradiation/Olaparib, showing that this phenotype seems to be dependent on another condition probably favored by low DPP9 levels. Control mice have only small amounts of tumor cells detected in lungs independent of the treatment condition. It was already shown that irradiation promotes metastasis formation in different cancer types in patients with head and neck cancer (29, 30), bladder cancer (29, 31) and uterine cervix cancer (29, 32). In breast cancer, mouse models in particular demonstrated this effect of increased metastatic burden upon irradiation (33, 34).

In summary, although local irradiation in combination with Olaparib treatment of MDA.MB.231-derived tumors with low DPP9 levels results in synthetic lethality (**Figure 7C**), potentially enhanced metastasis formation upon this treatment strategy might counteract the effect on tumor growth. Although DPP8/9 inhibition increased DNA damage in other TNBC cells as well, detailed analysis of these and other TNBC cell lines as well as patient-derived material is required to solidify the shown promising effects of irradiation therapy in combination with PARP inhibition on tumors with low DPP9 expression.

## Data Availability

The original contributions presented in the study are included in the article/**Supplementary Material**. Further inquiries can be directed to the corresponding author.
