# Supplementary material for: Reduced DPP9 levels sensitize experimental breast tumors to combinatory treatment with irradiation and Olaparib
Source: Front Oncol. 2026 Jun 10;16:1845048. doi: 10.3389/fonc.2026.1845048 (PMC13291933; doi:10.3389/fonc.2026.1845048)
Supplement: Supplementary file 1 [file DataSheet1.pdf]

Fig. S1

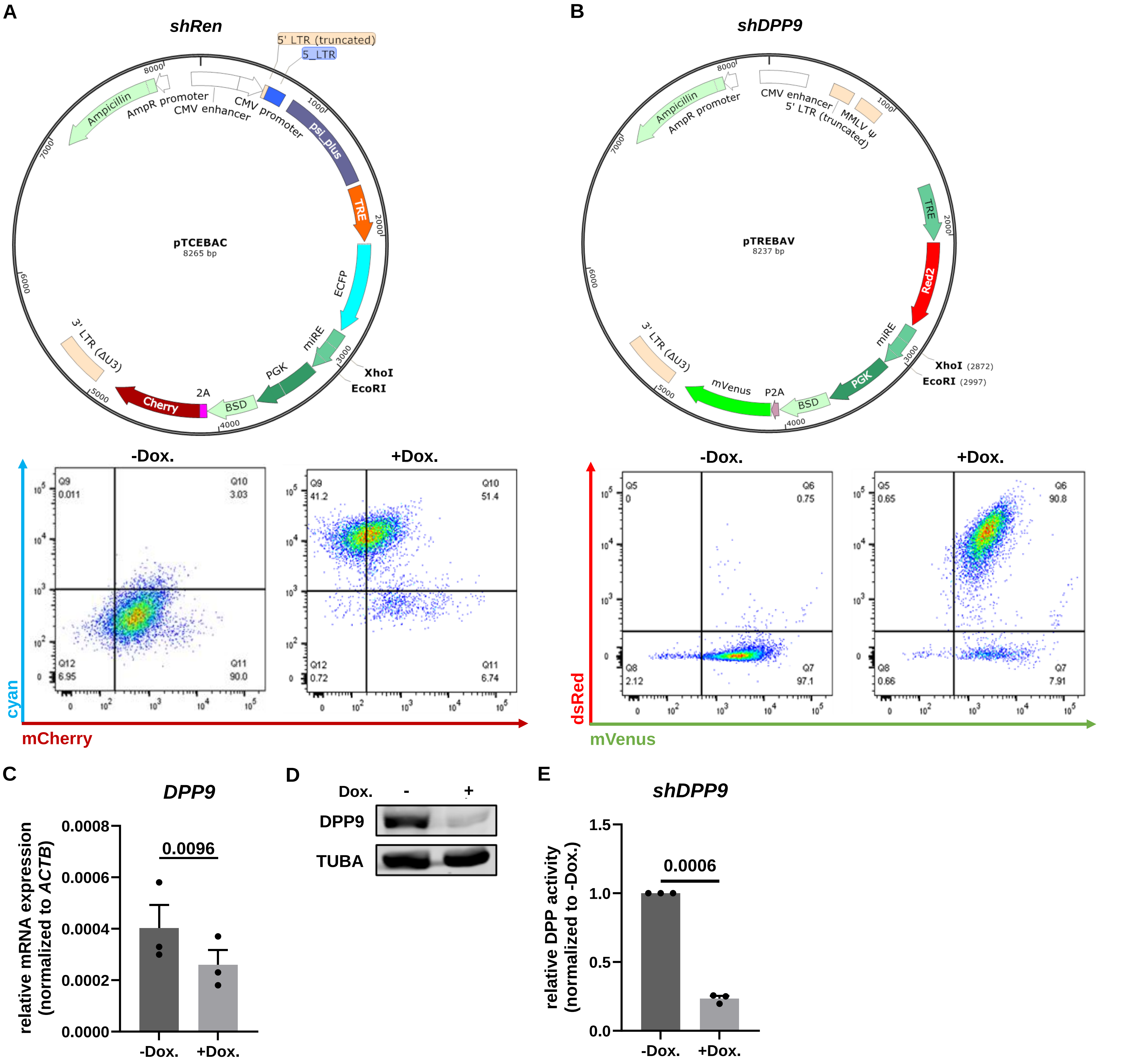

Figure S1 Validation of DPP9 knockdown system.

A, B Vector map of *shRen* in pTCEBAC (**A**) and *shDPP9* pTREBAV (**B**) showing encoded fluorscence labels anaylzed by flow cytometry. Constitutive fluorsecence is mCherry (**A**) or mVenus (**B**) and inducible fluorescence is cyan (**A**) or dsRed (**B**) for *shRen* or *shDPP9*, respectively. **C** *DPP9* mRNA expression in *shDPP9* MDA.MB.231 cells ± Doxycycline (Dox.). **D** DPP9 protein expression in *shDPP9* MDA.MB.231 cells ± Doxycycline (Dox.). **E** DPP activity in *shDPP9* MDA.MB.231 cells ± Doxycycline (Dox.). Bar graph shows mean +S.E.M and *p* value calculated by paired t-test (n=3 independent biological replicates).

Fig. S2

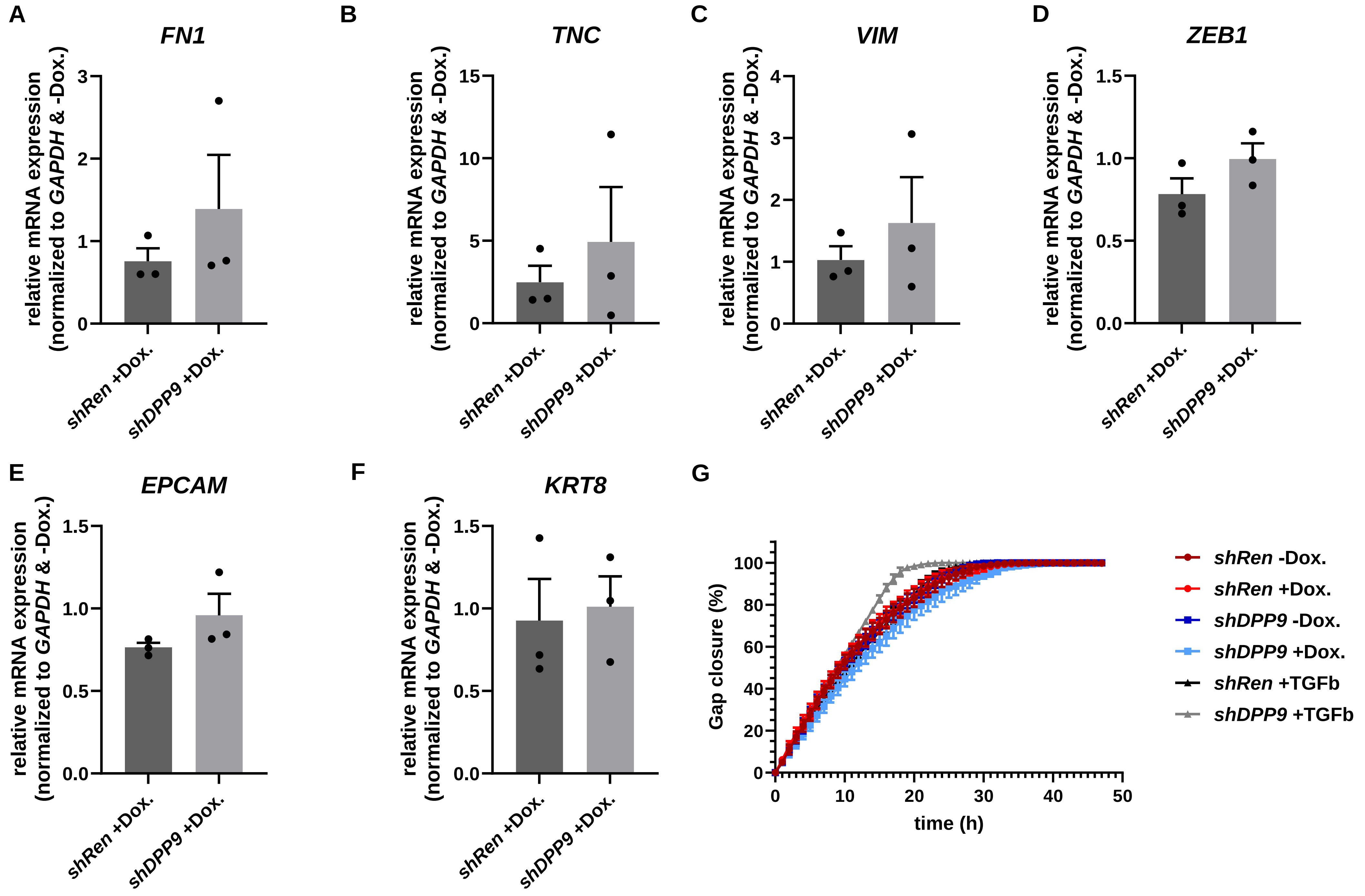

Figure S2 DPP9 deficiency had no impact on mesenchymal phenotype of MDA.MB.231 cells *in vitro*.
A-F mRNA expression of *FN1* (**A**), *TNC* (**B**), *VIM* (**C**), *ZEB1* (**D**), *EPCAM* (**E**) and *KRT8* (**F**) in *shRen* or *shDPP9* MDA.MB.231 cells ± Doxycycline (n=3 independent biological replicates). **G** Gap closure of *shRen* or *shDPP9* MDA.MB.231 cells ± Doxycycline or TGF-β (n=4 independent biological replicates). Bar and line graphs show mean +S.E.M and ±S.E.M., respectively.

Fig. S3

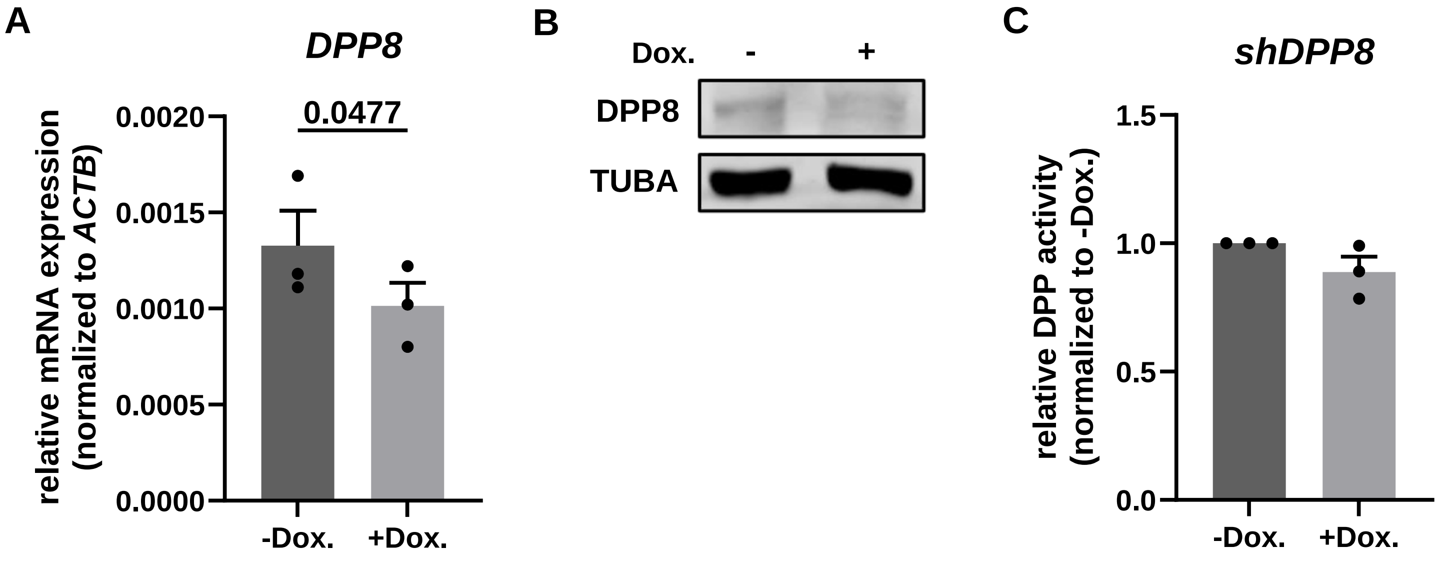

Figure S3 Validation of DPP8 knockdown system in pTCEBAC.
A *DPP8* mRNA expression in *shDPP8* MDA.MB.231 cells ± Doxycycline (Dox.). **B** DPP8 protein expression in *shDPP8* MDA.MB.231 cells ± Doxycycline (Dox.). **C** DPP activity assay in *shDPP8* MDA.MB.231 cells ± Doxycycline (Dox.). Bar graph shows mean +S.E.M and *p* value calculated by paired t-test (n=3 independent biological replicates).

Fig. S4

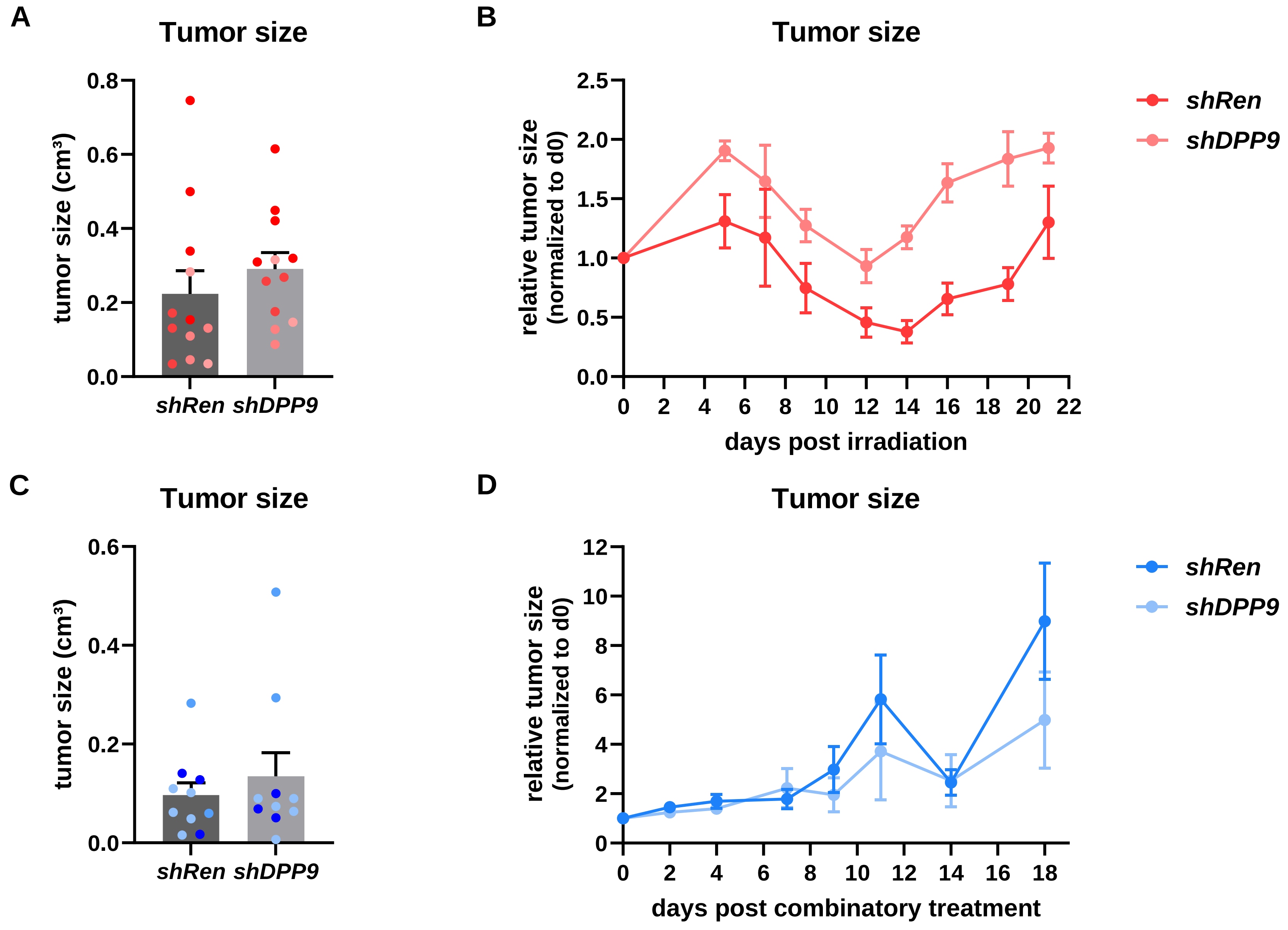

Figure S4 Tumor size at beginning of experiment and tumor size development during mouse experiment.
A,B Tumor size at start of irradiation (**A**) and representative tumor size of one experiment after irradiation during the mouse experiment (**B**). n=12 mice in 3 independent experiments. **C,D** Tumor size at start of combinatory treatment (**C**) and representative tumor size of one experiment after combinatory treatment during the mouse experiment (**D**). n=10 mice in 4 independent experiments. Bar and line graphs show mean +S.E.M and ±S.E.M., respectively.

qPCR raw data
from Figure 2, 3, S1, S2 and S3.

Figure 2

Replicate 1								
	Normoxia				Hypoxia			
	shRen		*shDPP9*		*shRen*		*shDPP9*	
Target	-Dox.	+Dox.	-Dox.	+Dox.	-Dox.	+Dox.	-Dox.	+Dox.
GAPDH (Houskeeping Gene)	21,13	20,28	20,27	20,11	20,15	20,66	21,05	22,27
	20,90	20,03	20,22	21,69	21,31	21,25	20,98	22,73
	20,62	20,63	20,34	20,25	20,09	20,71	21,80	22,85
Mean	20,88	20,31	20,28	20,68	20,52	20,87	21,28	22,62
Hif1a	25,89	25,78	25,66	25,69	26,03	26,5	26,36	26,98
	25,69	25,71	25,56	25,78	25,98	26,3	26,28	27,1
	25,24	25,54	25,66	25,68	26,00	26,29	26,63	27,43
Mean	25,61	25,68	25,63	25,72	26,00	26,36	26,42	27,17
DeltaCt (Norm. to GAPDH)	0,0379	0,0243	0,0245	0,0305	0,0223	0,0223	0,0282	0,0426
DeltaCt (Norm. to -Dox.)	1,00	0,64	1,00	1,25	1,00	1,00	1,00	1,51
VEGF	34,32	33,66	33,25	33,21	N/A	33,07	34,38	34,69
	33,65	33,67	33,4	33,17	N/A	33,49	33,85	34,22
	33,49	33,07	33,54	33,12	32,15	33,47	33,52	34,84
Mean	33,82	33,47	33,40	33,17	32,15	33,34	33,92	34,58
DeltaCt (Norm. to GAPDH)	0,000128	0,000110	0,000112	0,000175	0,000315	0,000176	0,000157	0,000250
DeltaCt (Norm. to -Dox.)	1,00	0,86	1,00	1,55	1,00	0,56	1,00	1,59
ANGPT1	32,17	31,71	32,38	32,40	32,32	32,16	32,75	31,25
	31,68	31,58	32,54	32,65	N/A	32,18	32,32	32,05
	31,90	31,13	32,63	N/A	N/A	32,47	32,20	31,55
Mean	31,92	31,47	32,52	32,53	32,32	32,27	32,42	31,62
DeltaCt (Norm. to GAPDH)	0,000477	0,000437	0,000207	0,000272	0,000280	0,000371	0,000441	0,001953
DeltaCt (Norm. to -Dox.)	1	0,91594529	1	1,31798444	1	1,32561944	1	4,42803513
CXCL1	27,03	27,73	28,77	29,23	N/A	27,63	30,84	31,14
	27,05	27,82	29,05	29,72	27,27	27,29	30,41	31,03
	26,98	27,74	28,99	N/A	26,79	27,56	30,69	31,51
Mean	27,02	27,76	28,94	29,48	27,03	27,49	30,65	31,23
DeltaCt (Norm. to GAPDH)	0,01421	0,00572	0,00247	0,00226	0,01095	0,01017	0,00151	0,00256
DeltaCt (Norm. to -Dox.)	1,00	0,40	1,00	0,91	1,00	0,93	1,00	1,69

	Normoxia				Hypoxia			
	shRen		*shDPP9*		*shRen*		*shDPP9*	
Target	-Dox.	+Dox.	-Dox.	+Dox.	-Dox.	+Dox.	-Dox.	+Dox.
GAPDH (Houskeeping Gene)	21,40	21,19	20,55	20,26	20,43	21,18	21,59	22,22
	21,88	20,94	20,47	20,39	21,01	21,34	21,60	22,93
	21,56	21,19	20,52	20,55	20,55	21,30	21,97	23,75
Mean	21,61	21,11	20,51	20,40	20,66	21,27	21,72	22,97
EGF	31,69	31,24	31,61	32,53	31,39	31,82	33,11	33,44
	31,43	31,28	31,58	32,18	31,47	31,61	32,41	33,5
	31,43	31,33	31,70	32,4	31,42	31,83	32,79	34,16
Mean	31,43	31,28	31,63	32,37	31,43	31,75	32,77	33,70
DeltaCt (Norm. to GAPDH)	0,001109	0,000864	0,000450	0,000249	0,000575	0,000700	0,000472	0,000587
DeltaCt (Norm. to -Dox.)	1,00	0,78	1,00	0,55	1,00	1,22	1,00	1,25
PIGF	28,43	27,47	27,74	27,44	27,69	27,27	27,64	27,94
	27,82	27,53	27,68	27,42	27,73	27,33	27,46	27,65
	27,68	27,56	27,79	27,50	27,72	27,34	27,96	28,07
Mean	27,98	27,52	27,74	27,45	27,71	27,31	27,69	27,89
DeltaCt (Norm. to GAPDH)	0,01215	0,01173	0,00669	0,00753	0,00755	0,01520	0,01599	0,03303
DeltaCt (Norm. to -Dox.)	1,00	0,97	1,00	1,13	1,00	2,01	1,00	2,07

Figure 2

Replicate 2								
	Normoxia				Hypoxia			
	shRen		*shDPP9*		*shRen*		*shDPP9*	
**Target**	**-Dox.**	**+Dox.**	**-Dox.**	**+Dox.**	**-Dox.**	**+Dox.**	**-Dox.**	**+Dox.**
GAPDH (Houskeeping Gene)	21,64	21,3	21,05	21,02	20,06	20,24	20,37	21,53
	21,92	21,19	20,76	21,12	20,02	20,29	20,21	21,89
	21,58	21,19	20,61	21,15	20,14	20,24	20,46	22,31
**Mean**	21,71	21,23	20,81	21,10	20,07	20,26	20,35	21,91
Hif1a	27,45	27,07	27,07	27,21	26,59	26,85	27,14	27,89
	27,06	27,16	27,16	27,17	26,6	26,83	26,95	27,96
	26,81	26,92	27,32	27,26	26,62	26,69	27,2	28,27
**Mean**	27,11	27,05	27,18	27,21	26,60	26,79	27,10	28,04
**DeltaCt (Norm. to GAPDH)**	0,0238	0,0177	0,0120	0,0144	0,0108	0,0108	0,0093	0,0143
**DeltaCt (Norm. to -Dox.)**	1,00	0,74	1,00	1,20	1,00	1,00	1,00	1,54
VEGF	35,90	35,44	34,59	34,27	32,65	33,42	34,61	34,57
	34,76	35,54	34,35	34,26	32,91	33,78	33,9	34,63
	35,46	35,02	34,63	34,03	33,16	33,47	34,19	34,77
**Mean**	35,37	35,33	34,52	34,19	32,91	33,56	34,23	34,66
**DeltaCt (Norm. to GAPDH)**	0,000077	0,000057	0,000074	0,000115	0,000137	0,000099	0,000066	0,000146
**DeltaCt (Norm. to -Dox.)**	1,00	0,73	1,00	1,54	1,00	0,72	1,00	2,20
ANGPT1	34,33	33,72	33,73	33,2	33,66	32,72	34,21	33,15
	33,50	33,50	33,67	33,61	33,29	33,32	33,44	33,24
	34,18	33,33	34,04	33,95	33,97	32,99	33,06	33,35
**Mean**	34,00	33,52	33,81	33,59	33,64	33,01	33,57	33,25
**DeltaCt (Norm. to GAPDH)**	0,000200	0,000200	0,000122	0,000174	0,000082	0,000145	0,000105	0,000387
**DeltaCt (Norm. to -Dox.)**	1,00	1,00	1,00	1,43	1,00	1,76	1,00	3,70
CXCL1	29,20	28,49	31,07	31,32	26,36	26,45	30,94	32,4
	29,09	28,55	31,12	31,35	26,33	26,51	30,78	32,31
	28,98	28,35	31,27	31,33	26,29	26,53	30,88	32,46
**Mean**	29,09	28,46	31,15	31,33	26,33	26,50	30,87	32,39
**DeltaCt (Norm. to GAPDH)**	0,00602	0,00663	0,00077	0,00083	0,01311	0,01323	0,00068	0,00070
**DeltaCt (Norm. to -Dox.)**	1,00	1,10	1,00	1,08	1,00	1,01	1,00	1,03

	Normoxia				Hypoxia			
	shRen		*shDPP9*		*shRen*		*shDPP9*	
**Target**	**-Dox.**	**+Dox.**	**-Dox.**	**+Dox.**	**-Dox.**	**+Dox.**	**-Dox.**	**+Dox.**
GAPDH (Houskeeping Gene)	22,81	22,46	21,44	21,78	20,80	21,44	20,99	22,29
	23,07	22,04	21,59	22,27	21,37	21,14	21,29	22,65
	22,54	22,54	21,44	21,85	20,59	20,89	21,77	23,75
**Mean**	22,81	22,35	21,49	21,97	20,92	21,16	21,35	22,90
EGF	33,79	32,27	33,62	33,17	32,19	32,60	33,67	33,46
	33,45	32,51	33,03	32,75	32,61	32,58	33,14	33,24
	33,24	32,47	32,99	32,44	32,06	32,27	33,43	34,02
**Mean**	33,35	32,42	33,21	32,79	32,29	32,48	33,41	33,57
**DeltaCt (Norm. to GAPDH)**	0,000672	0,000930	0,000296	0,000553	0,000379	0,000389	0,000234	0,000611
**DeltaCt (Norm. to -Dox.)**	1,00	1,38	1,00	1,87	1,00	1,03	1,00	2,61
PIGF	29,69	28,56	28,42	28,40	28,58	28,22	28,21	29,2
	29,22	28,75	28,21	28,55	28,67	28,2	28,05	29,29
	29,06	28,96	28,87	28,67	28,87	28,4	28,28	29,73
**Mean**	29,32	28,76	28,50	28,54	28,71	28,27	28,18	29,41
**DeltaCt (Norm. to GAPDH)**	0,01092	0,01176	0,00776	0,01050	0,00453	0,00721	0,00879	0,01097
**DeltaCt (Norm. to -Dox.)**	1,00	1,08	1,00	1,35	1,00	1,59	1,00	1,25

Figure 2

Replicate 3								
	Normoxia				Hypoxia			
	shRen		*shDPP9*		*shRen*		*shDPP9*	
Target	-Dox.	+Dox.	-Dox.	+Dox.	-Dox.	+Dox.	-Dox.	+Dox.
GAPDH (Houskeeping Gene)	21,19	20,72	20,48	20,6	19,75	20,08	20,49	21,49
	21,22	20,44	20,49	20,66	20,02	19,88	20,45	21,83
	20,86	20,52	20,51	20,7	19,6	20,3	20,69	22,03
Mean	21,09	20,56	20,49	20,65	19,79	20,09	20,54	21,78
Hif1a	25,22	25,11	24,99	23,56	25,03	25,49	25,61	26,01
	25,00	24,96	25,04	23,53	25,05	25,21	25,41	26,24
	24,53	25,1	25,14	23,3	25,28	25,19	25,64	26,39
Mean	24,92	25,06	25,06	23,46	25,12	25,30	25,55	26,21
DeltaCt (Norm. to GAPDH)	0,0705	0,0443	0,0423	0,1426	0,0249	0,0270	0,0310	0,0464
DeltaCt (Norm. to -Dox.)	1,00	0,63	1,00	3,37	1,00	1,09	1,00	1,49
VEGF	33,69	33,53	33,8	33,77	33	33,31	33,46	33,33
	32,95	33,8	33,44	33,83	32,98	33,4	32,99	33,33
	33,31	33,36	33,63	33,59	32,97	33,45	32,85	34,22
Mean	33,32	33,56	33,62	33,73	32,98	33,39	33,10	33,63
DeltaCt (Norm. to GAPDH)	0,000209	0,000122	0,000112	0,000116	0,000107	0,000099	0,000166	0,000272
DeltaCt (Norm. to -Dox.)	1,00	0,58	1,00	1,04	1,00	0,93	1,00	1,64
ANGPT1	31,76	31,53	32,01	30,67	31,81	31,22	32,49	31,6
	31,29	31,47	32,03	30,75	31,95	31,39	32,13	31,29
	31,74	31,52	32,1	30,88	31,63	31,48	32,04	31,78
Mean	31,60	31,51	32,05	30,77	31,80	31,36	32,22	31,56
DeltaCt (Norm. to GAPDH)	0,000687	0,000507	0,000333	0,000903	0,000243	0,000403	0,000305	0,001143
DeltaCt (Norm. to -Dox.)	1,00	0,74	1,00	2,71	1,00	1,66	1,00	3,74
CXCL1	27,03	27,2	29,17	29,07	26,47	26,23	29,5	29,68
	26,76	27,08	29,2	29,21	26,49	26,33	29,34	29,54
	26,96	27,22	29,16	29,06	26,59	26,38	29,59	29,95
Mean	26,92	27,17	29,18	29,11	26,52	26,31	29,48	29,72
DeltaCt (Norm. to GAPDH)	0,01762	0,01026	0,00243	0,00284	0,00944	0,01335	0,00205	0,00407
DeltaCt (Norm. to -Dox.)	1,00	0,58	1,00	1,17	1,00	1,41	1,00	1,99

	Normoxia				Hypoxia			
	shRen		*shDPP9*		*shRen*		*shDPP9*	
Target	-Dox.	+Dox.	-Dox.	+Dox.	-Dox.	+Dox.	-Dox.	+Dox.
GAPDH (Houskeeping Gene)	22,14	20,86	20,58	21,67	20,14	20,34	20,63	22,77
	21,47	20,69	20,8	22,16	20,15	20,27	20,69	21,38
	21,36	20,53	20,74	21,63	20,02	20,34	21,16	22,25
Mean	21,66	20,69	20,71	21,82	20,10	20,32	20,83	22,13
EGF	31,2	30,53	30,79	32,12	30,85	30,73	31,7	31,56
	31,28	30,29	31,45	32,47	31,19	30,97	31,76	32,03
	30,68	30,45	31,03	32,24	31,01	30,9	32,09	32,25
Mean	30,98	30,42	31,09	32,28	31,02	30,87	31,85	31,95
DeltaCt (Norm. to GAPDH)	0,001561	0,001178	0,000749	0,000712	0,000519	0,000667	0,000480	0,001111
DeltaCt (Norm. to -Dox.)	1,00	0,75	1,00	0,95	1,00	1,29	1,00	2,31
PIGF	28,88	27,44	27,51	28,42	27,94	27,67	27,99	27,71
	28,36	27,77	27,46	28,29	28,17	27,83	27,77	27,89
	28,21	27,96	27,59	28,43	28,31	27,87	28,05	28,02
Mean	28,48	27,72	27,52	28,38	28,14	27,79	27,94	27,87
DeltaCt (Norm. to GAPDH)	0,00881	0,00765	0,00889	0,01060	0,00381	0,00563	0,00724	0,01871
DeltaCt (Norm. to -Dox.)	1,00	0,87	1,00	1,19	1,00	1,48	1,00	2,58

Figure 3

	shRen				*shDPP9*		
**Target**	**#1**	**#2**	**#3**	**#4**	**#1**	**#3**	**#4**
GAPDH (Houskeeping Gene)	22,38	24,73	22,18	30,69	28,51	26,33	33,75
	22,51	24,6	22,38	31,4	28,65	26,42	35,38
	22,14	24,57	21,74	31,17	28,55	26,78	35,23
**Mean**	22,34	24,63	22,10	31,09	28,57	26,51	34,79
**FN**	23,26	26,07	23,38	30,67	30,26	28,37	34,75
	22,85	26,3	23,45	30,53	30,61	28,35	34,37
	22,63	25,98	23,5	30,61	30,41	28,51	34,84
**Mean**	22,91	26,12	23,44	30,60	30,43	28,41	34,65
**DeltaCt (Norm. to GAPDH)**	0,6736	0,3577	0,3941	1,3980	0,2761	0,2679	1,0968
**TNC**	29,23	32,52	29,38	37,34	37,35	33,68	37,53
	28,94	31,62	29,39	N/A	37,43	35,15	N/A
	28,95	33,05	29,4	N/A	37,38	34,25	N/A
**Mean**	29,04	32,40	29,39	37,34	37,39	34,36	37,53
**DeltaCt (Norm. to GAPDH)**	0,009641	0,004603	0,006390	0,013109	0,002218	0,004334	0,149339
**VIM**	21,18	25,86	21,75	33,18	30,39	26,49	35,51
	20,66	25,93	21,77	32,79	30,43	26,39	35,82
	20,97	25,79	21,85	33,16	30,4	26,64	N/A
**Mean**	20,94	25,86	21,79	33,04	30,41	26,51	35,67
**DeltaCt (Norm. to GAPDH)**	2,651239	0,427304	1,239708	0,257623	0,279968	1,002313	0,543996
**Epcam**	27,99	28,36	27,67	32,11	30,71	32,28	38,5
	27,43	28,55	27,58	32,12	31,35	32,23	35,93
	27,25	28,61	27,74	32,21	30,98	32,2	36,13
**Mean**	27,56	28,51	27,66	32,15	31,01	32,24	36,85
**DeltaCt (Norm. to GAPDH)**	0,02695	0,06824	0,02115	0,47963	0,18386	0,01888	0,23871

	shRen				*shDPP9*		
**Target**	**#1**	**#2**	**#3**	**#4**	**#1**	**#3**	**#4**
GAPDH (Houskeeping Gene)	23,21	25,77	22,65	31,16	29,69	26,98	35,2
	22,87	25,37	23	31,17	28,95	27,12	35,05
	22,93	25,45	23,01	30,88	29,85	27,15	35,6
**Mean**	23,00	25,53	22,89	31,07	29,50	27,08	35,28
**ZEB1**	26,74	28,58	27,21	31,08	31,56	30,28	33,56
	26,57	28,28	27,26	31,27	31,53	29,98	32,68
	26,55	28,42	27,40	31,01	31,58	30,2	33,52
**Mean**	26,62	28,43	27,29	31,12	31,56	30,15	33,25
**DeltaCt (Norm. to GAPDH)**	0,081522	0,134282	0,047257	0,965936	0,239816	0,119080	4,084049
**KRT5**	N/A	36,57	36,61	N/A	N/A	N/A	N/A
	N/A	37,51	36,13	N/A	N/A	N/A	N/A
	37,35	N/A	34,88	N/A	44,6	37,44	N/A
**Mean**	37,35	37,04	35,87	0,00	44,60	37,44	0,00
**DeltaCt (Norm. to GAPDH)**	0,00005	0,00034	0,00012	0,00000	0,00003	0,00076	0,00000

Figure S1

Replicate 1		
	shDPP9	
Target	-Dox.	+Dox.
ACTB (Houskeeping Gene)	19,59	20,31
	20,01	20,21
	20,03	20,37
Mean	19,88	20,29
DPP9	30,48	31,52
	30,64	31,67
	30,79	31,89
Mean	30,64	31,70
DeltaCt (Norm. to GAPDH)	0,000578	0,000369

Replicate 2		
	shDPP9	
Target	-Dox.	+Dox.
ACTB (Houskeeping Gene)	18,14	18,50
	18,29	18,68
	18,70	19,32
Mean	18,37	18,83
DPP9	30,25	31,38
	30,10	31,29
	29,89	31,25
Mean	30,08	31,31
DeltaCt (Norm. to GAPDH)	0,000299	0,000175

Replicate 3		
	shDPP9	
Target	-Dox.	+Dox.
ACTB (Houskeeping Gene)	17,78	18,35
	18,04	18,54
	18,16	19,01
Mean	17,99	18,64
DPP9	29,43	30,52
	29,60	30,55
	29,70	31,05
Mean	29,58	30,71
DeltaCt (Norm. to GAPDH)	0,000326	0,000233

Figure S2

Replicate 1				
	shRen		*shDPP9*	
**Target**	**-Dox.**	**+Dox.**	**-Dox.**	**+Dox.**
GAPDH (Houskeeping Gene)	21,13	20,28	20,27	20,11
	20,90	20,03	20,22	21,69
	20,62	20,63	20,34	20,25
**Mean**	20,88	20,31	20,28	20,68
FN1	22,91	23,08	23,71	24,5
	22,72	22,87	23,65	24,33
	22,48	22,65	23,53	24,44
**Mean**	22,70	22,87	23,63	24,42
**DeltaCt (Norm. to GAPDH)**	0,2832	0,1704	0,0978	0,0748
**DeltaCt (Norm. to -Dox.)**	1,00	0,60	1,00	0,76
TNC	28,14	26,93	27,75	27,73
	27,96	26,87	27,7	31,71
	28,05	26,92	27,9	28,28
**Mean**	28,05	26,91	27,78	29,24
**DeltaCt (Norm. to GAPDH)**	0,006960	0,010356	0,005499	0,002656
**DeltaCt (Norm. to -Dox.)**	1,00	1,49	1,00	0,48
VIM	20,08	19,4	19,88	20,28
	19,64	19,77	19,8	19,93
	19,56	19,57	20	22,92
**Mean**	19,76	19,58	19,89	21,04
**DeltaCt (Norm. to GAPDH)**	2,178497	1,662476	1,304352	0,779165
**DeltaCt (Norm. to -Dox.)**	1	0,7631296	1	0,59735757
Epcam	27,65	27,58	26,7	27,01
	27,56	27,59	26,75	26,85
	27,34	27,12	26,98	N/A
**Mean**	27,52	27,43	26,81	26,93
**DeltaCt (Norm. to GAPDH)**	0,01007	0,00721	0,01080	0,01317
**DeltaCt (Norm. to -Dox.)**	1,00	0,72	1,00	1,22

	shRen		*shDPP9*	
**Target**	**-Dox.**	**+Dox.**	**-Dox.**	**+Dox.**
GAPDH (Houskeeping Gene)	21,40	21,19	20,55	20,26
	21,88	20,94	20,47	20,39
	21,56	21,19	20,52	20,55
**Mean**	21,61	21,11	20,51	20,40
ZEB1	25,2	25	25,27	25,37
	24,81	24,88	25,32	25,49
	25,04	25,11	25,27	25,44
**Mean**	25,02	25,00	25,29	25,43
**DeltaCt (Norm. to GAPDH)**	0,094514	0,067452	0,036567	0,030536
**DeltaCt (Norm. to -Dox.)**	1,00	0,71	1,00	0,84
KRT8	30,75	30,22	30,35	30,66
	30,11	30,26	30,08	30,64
	30,09	30,38	30,3	30,79
**Mean**	30,32	30,29	30,24	30,70
**DeltaCt (Norm. to GAPDH)**	0,00240	0,00172	0,00118	0,00080
**DeltaCt (Norm. to -Dox.)**	1,00	0,72	1,00	0,68

Figure S2

Replicate 2				
	shRen		*shDPP9*	
**Target**	**-Dox.**	**+Dox.**	**-Dox.**	**+Dox.**
GAPDH (Houskeeping Gene)	21,64	21,3	21,05	21,02
	21,92	21,19	20,76	21,12
	21,58	21,19	20,61	21,15
**Mean**	21,71	21,23	20,81	21,10
FN1	25,1	24,26	25,27	26,1
	24,85	24,3	25,33	26,09
	24,63	24,27	25,43	26,22
**Mean**	24,86	24,28	25,34	26,14
**DeltaCt (Norm. to GAPDH)**	0,1129	0,1207	0,0431	0,0304
**DeltaCt (Norm. to -Dox.)**	1,00	1,07	1,00	0,71
TNC	30,58	27,89	29,08	27,83
	30,62	28,04	28,82	27,66
	30,86	28,14	29	27,72
**Mean**	30,69	28,02	28,97	27,74
**DeltaCt (Norm. to GAPDH)**	0,001990	0,008995	0,003496	0,010027
**DeltaCt (Norm. to -Dox.)**	1,00	4,52	1,00	2,87
VIM	22,46	21,05	21,18	21,24
	22,12	21,18	21,05	21,12
	22,01	21,23	21,33	21,21
**Mean**	22,20	21,15	21,19	21,19
**DeltaCt (Norm. to GAPDH)**	0,715323	1,052145	0,768438	0,937354
**DeltaCt (Norm. to -Dox.)**	1,00	1,47	1,00	1,22
Epcam	28,48	28,32	27	27,93
	28,39	28,11	27,26	27,57
	28,2	28,07	27,4	27,77
**Mean**	28,36	28,17	27,22	27,76
**DeltaCt (Norm. to GAPDH)**	0,01000	0,00814	0,01173	0,00989
**DeltaCt (Norm. to -Dox.)**	1,00	0,81	1,00	0,84

	shRen		*shDPP9*	
**Target**	**-Dox.**	**+Dox.**	**-Dox.**	**+Dox.**
GAPDH (Houskeeping Gene)	22,81	22,46	21,44	21,78
	23,07	22,04	21,59	22,27
	22,54	22,54	21,44	21,85
**Mean**	22,81	22,35	21,49	21,97
ZEB1	27,15	26,25	26,52	26,68
	26,73	26,3	26,42	26,86
	26,6	26,68	26,54	26,72
**Mean**	26,83	26,41	26,49	26,75
**DeltaCt (Norm. to GAPDH)**	0,061640	0,059816	0,031178	0,036230
**DeltaCt (Norm. to -Dox.)**	1,00	0,97	1,00	1,16
KRT8	32,91	31,88	30,95	30,68
	32,64	31,4	30,76	30,98
	32,38	31,73	31,12	31,43
**Mean**	32,64	31,67	30,94	31,03
**DeltaCt (Norm. to GAPDH)**	0,00109	0,00156	0,00143	0,00187
**DeltaCt (Norm. to -Dox.)**	1,00	1,43	1,00	1,31

Figure S2

Replicate 3				
	shRen		*shDPP9*	
**Target**	**-Dox.**	**+Dox.**	**-Dox.**	**+Dox.**
GAPDH (Houskeeping Gene)	21,19	20,72	20,48	20,6
	21,22	20,44	20,49	20,66
	20,86	20,52	20,51	20,7
**Mean**	21,09	20,56	20,49	20,65
**FN1**	23,45	23,61	24,15	23
	23,20	23,27	24,19	23,05
	22,9	23,3	24,18	22,65
**Mean**	23,18	23,39	24,17	22,90
**DeltaCt (Norm. to GAPDH)**	0,2343	0,1403	0,0780	0,2107
**DeltaCt (Norm. to -Dox.)**	1,00	0,60	1,00	2,70
**TNC**	28,11	26,57	27,8	24,34
	27,36	26,7	27,56	24,32
	27,51	26,6	27,75	24,38
**Mean**	27,66	26,62	27,70	24,35
**DeltaCt (Norm. to GAPDH)**	0,010525	0,014954	0,006754	0,077303
**DeltaCt (Norm. to -Dox.)**	1,00	1,42	1,00	11,45
**VIM**	19,58	18,37	19,64	18,09
	19,01	19,1	19,56	18,23
	19,01	19,24	19,71	18,22
**Mean**	19,20	18,90	19,64	18,18
**DeltaCt (Norm. to GAPDH)**	3,706352	3,152872	1,810850	5,553254
**DeltaCt (Norm. to -Dox.)**	1,00	0,85	1,00	3,07
**Epcam**	27,84	27,57	26,34	26,76
	27,67	27,58	26,6	26,9
	27,44	27,39	26,54	27,18
**Mean**	27,65	27,51	26,49	26,95
**DeltaCt (Norm. to GAPDH)**	0,01060	0,00807	0,01563	0,01275
**DeltaCt (Norm. to -Dox.)**	1,00	0,76	1,00	0,82

	shRen		*shDPP9*	
**Target**	**-Dox.**	**+Dox.**	**-Dox.**	**+Dox.**
GAPDH (Houskeeping Gene)	22,14	20,86	20,58	21,67
	21,47	20,69	20,8	22,16
	21,36	20,53	20,74	21,63
**Mean**	21,66	20,69	20,71	21,82
**ZEB1**	25,86	25,14	25,28	26,35
	25,32	25,11	25,25	26,44
	25,6	25,41	25,54	26,66
**Mean**	25,59	25,22	25,36	26,48
**DeltaCt (Norm. to GAPDH)**	0,065305	0,043385	0,039830	0,039464
**DeltaCt (Norm. to -Dox.)**	1,00	0,66	1,00	0,99
**KRT8**	30,22	29,5	29,9	30,63
	29,71	29,76	29,65	31,09
	30,04	29,79	29,97	30,94
**Mean**	29,99	29,68	29,84	30,89
**DeltaCt (Norm. to GAPDH)**	0,00310	0,00197	0,00178	0,00186
**DeltaCt (Norm. to -Dox.)**	1,00	0,63	1,00	1,05

Figure S3

Replicate 1		
	shDPP8	
Target	-Dox.	+Dox.
ACTB (Houskeeping Gene)	20,15	20,62
	20,13	20,62
	19,96	20,86
Mean	20,08	20,70
DPP8	29,91	30,59
	29,78	30,55
	29,72	30,76
Mean	29,81	30,63
DeltaCt (Norm. to GAPDH)	0,00118	0,00102

Replicate 2		
	shDPP8	
Target	-Dox.	+Dox.
ACTB (Houskeeping Gene)	17,78	18,46
	18,09	18,98
	18,36	18,70
Mean	18,08	18,71
DPP8	27,71	29,03
	27,78	28,82
	28,16	29,11
Mean	27,89	28,99
DeltaCt (Norm. to GAPDH)	0,00111	0,00080

Replicate 3		
	shDPP8	
Target	-Dox.	+Dox.
ACTB (Houskeeping Gene)	17,43	18,22
	17,80	18,16
	17,67	18,85
Mean	17,63	18,41
DPP8	26,67	27,93
	26,90	28,20
	26,95	28,15
Mean	26,84	28,09
DeltaCt (Norm. to GAPDH)	0,00169	0,00122

Western Blot raw data
from Figure 3, 4, S1 and S3.

Figure 3

Target	shRen				shDPP9			
	#1	#2	#3	#4	#1	#2	#3	#4
VIM	46200	58900	60400	49600	52700	44100	53600	40100
GAPDH (housekeeping gene)	12800	17000	7920	9600	13400	14900	10600	13700
Normalized to GAPDH	3,609375	3,46470588	7,62626263	5,16666667	3,93283582	2,95973154	5,05660377	2,9270073
KRT5	62100	51700	42000	52400	56200	50000	91900	26500
GAPDH (housekeeping gene)	51300	45400	40300	49600	65000	62200	58200	71300
Normalized to GAPDH	1,21052632	1,13876652	1,04218362	1,05645161	0,86461538	0,80385852	1,5790378	0,371669

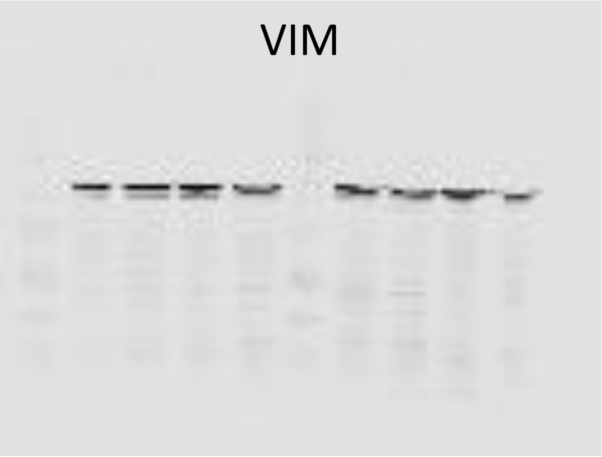

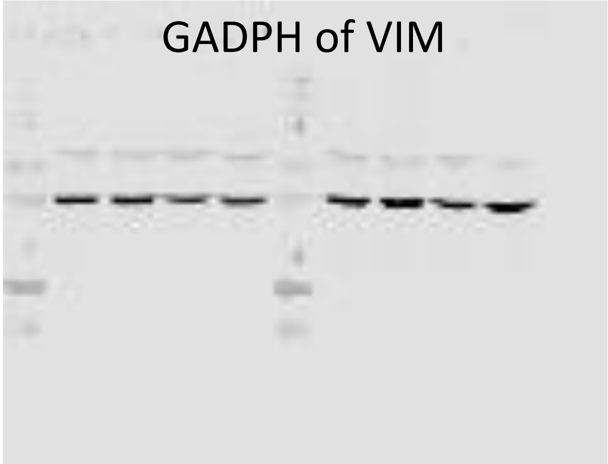

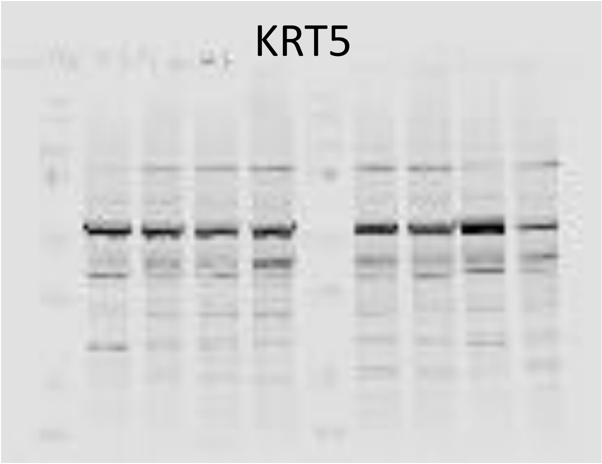

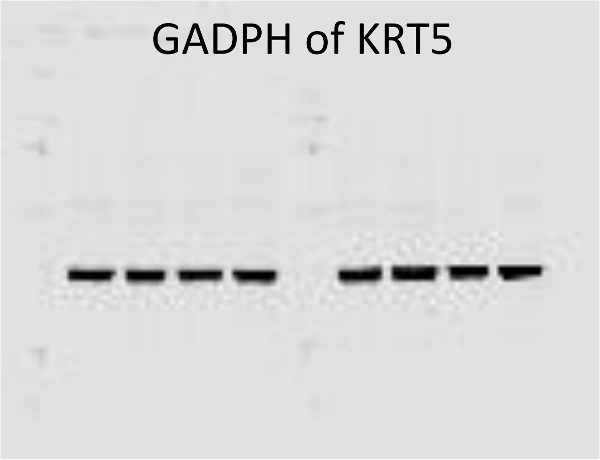

Samples from table from left to right displayed in Western Blots.

Figure 4

Replicate 1				
Target	shDPP8		shDPP9	
	-Dox.	+Dox.	-Dox.	+Dox.
γ-H2A.X	77036402	65624254	67865820	98987289
H2A.X	102359528	98393727	101596999	115607073
Normalized to H2A.X	0,75260607	0,66695567	0,6679904	0,85623904
Normalized to -Dox.	1	0,88619491	1	1,28181339

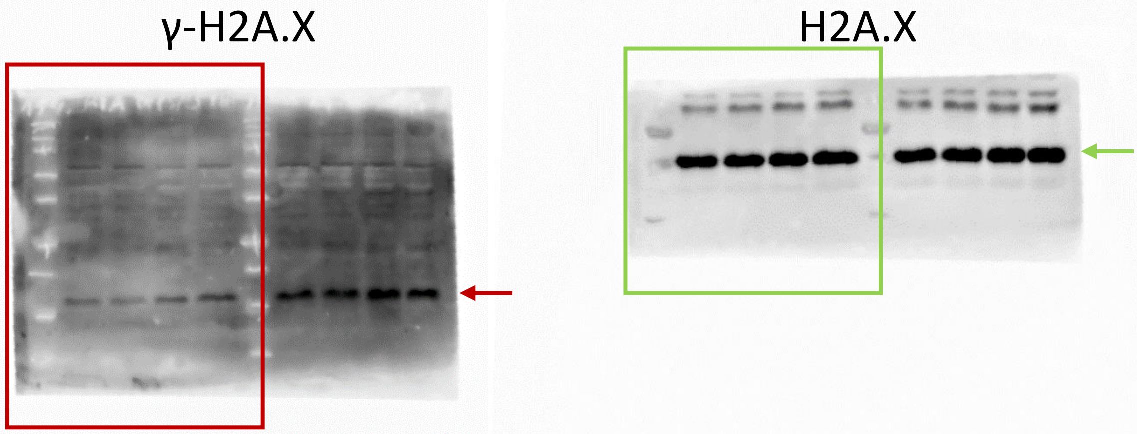

Replicate 2 (representative Western Blot shown in Fig. 4B)				
Target	shDPP8		shDPP9	
	-Dox.	+Dox.	-Dox.	+Dox.
γ-H2A.X	69280683	56525842	61814004	68272949
H2A.X	73496437	67081836	66900500	58401442
Normalized to H2A.X	0,94264002	0,84264006	0,92396924	1,16902848
Normalized to -Dox.	1	0,893915	1	1,26522446

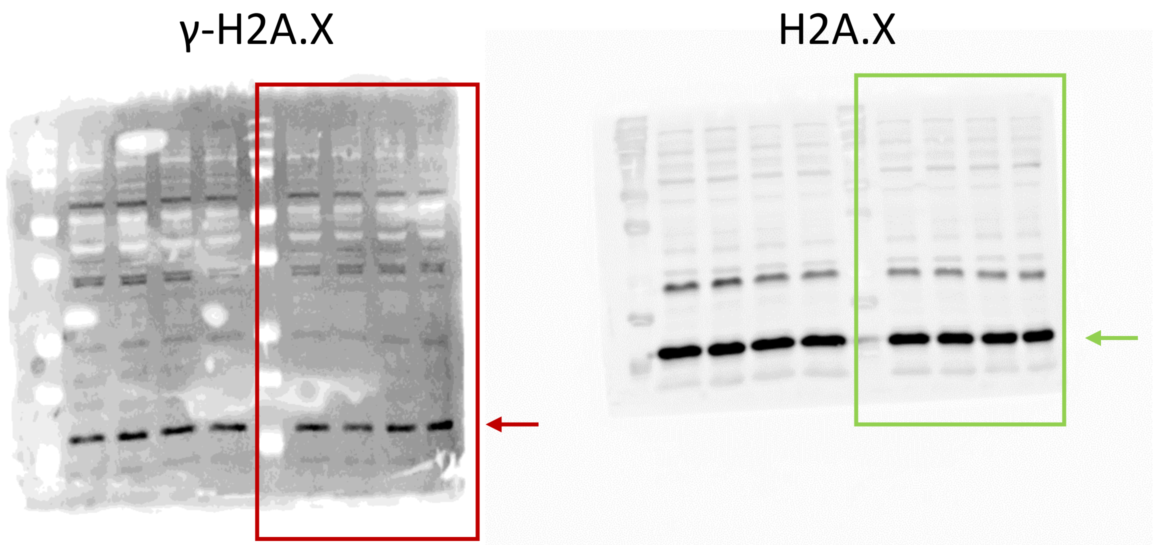

Samples from table from left to right displayed in Western Blots.
Box highlights samples used for quantification.

Figure 4

Replicate 3				
Target	shDPP8		shDPP9	
	-Dox.	+Dox.	-Dox.	+Dox.
γ-H2A.X	9553935	10517562	42454694	38615622
H2A.X	45613873	50749691	64824769	59322736
Normalized to H2A.X	0,2094524	0,20724386	0,6549147	0,65094135
Normalized to -Dox.	1	0,98945568	1	0,99393303

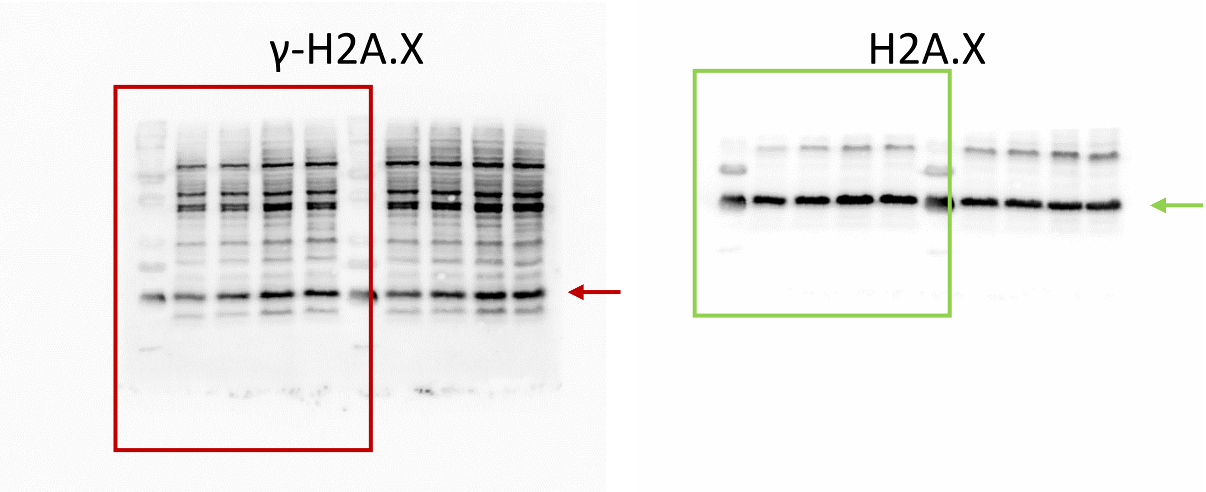

Replicate 4				
Target	shDPP8		shDPP9	
	-Dox.	+Dox.	-Dox.	+Dox.
γ-H2A.X	69280683	56525842	61814004	68272949
H2A.X	46279770	36099235	49286985	42442399
Normalized to H2A.X	1,49699713	1,56584598	1,25416485	1,6086025
Normalized to -Dox.	1	1,0459913	1	1,2826085

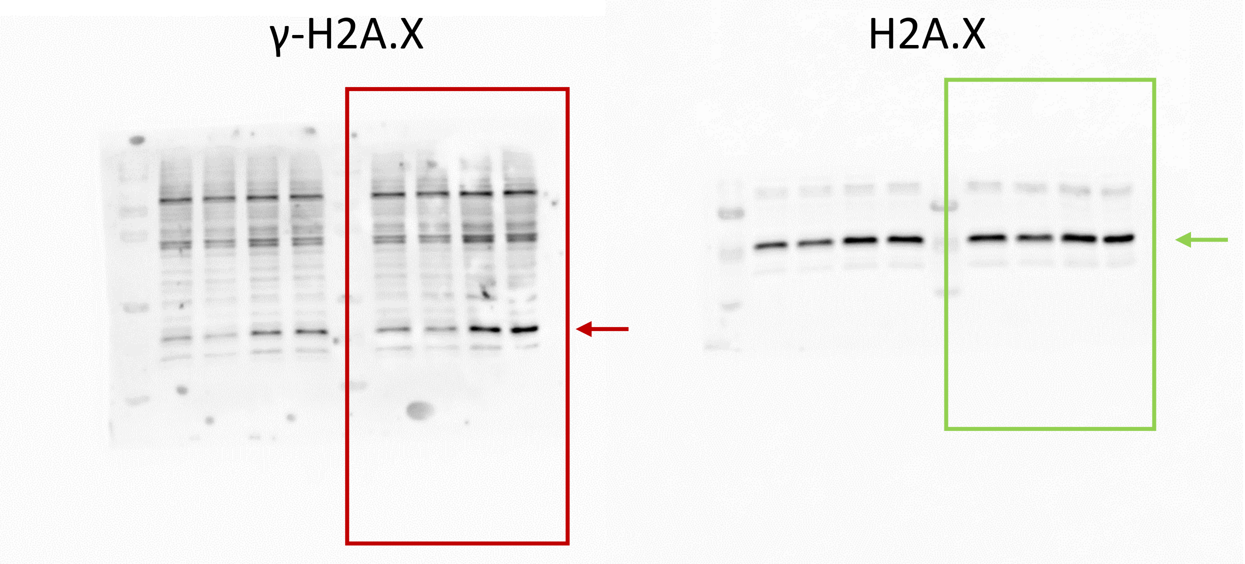

Samples from table from left to right displayed in Western Blots.
Box highlights samples used for quantification.

Figure S1

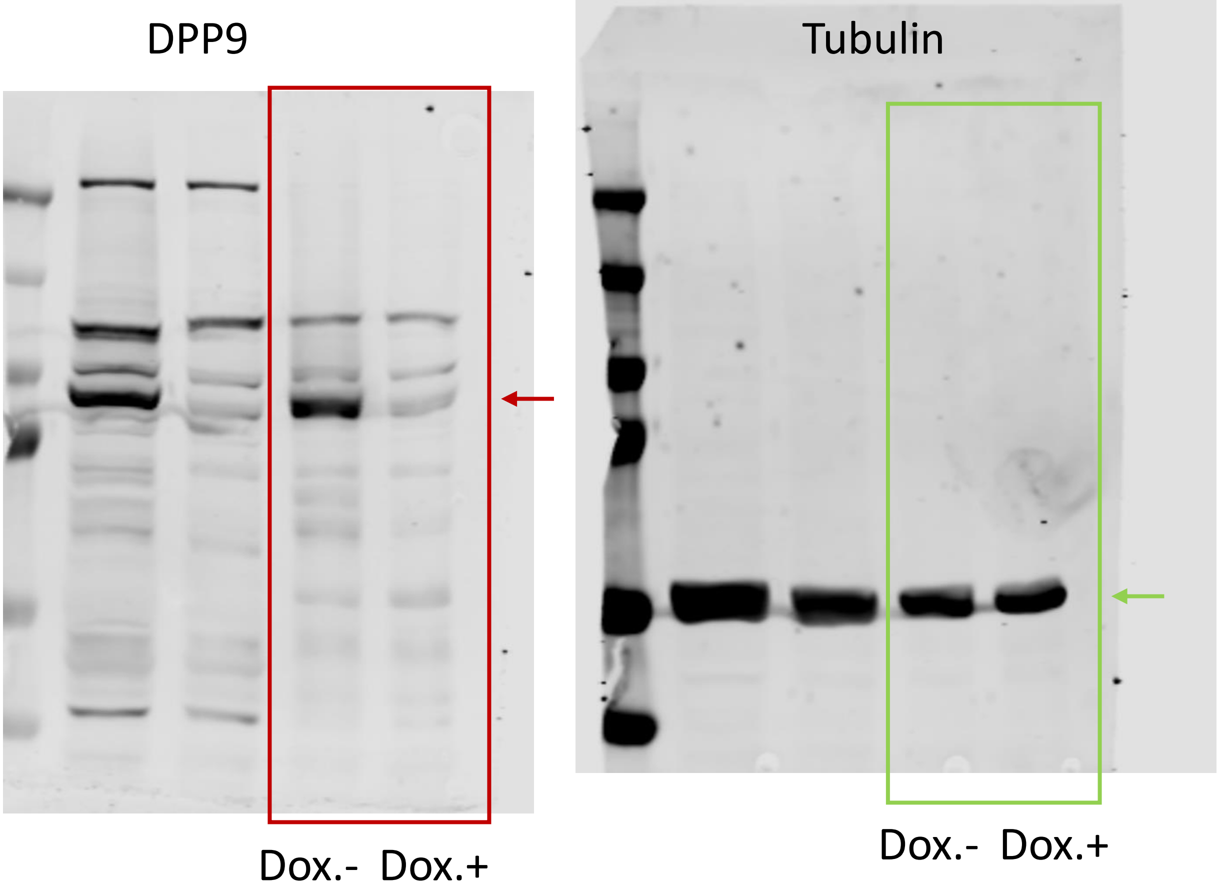

1 replicate is shown in the graph without quantification.
(Quantification of enzyme activity is shown)
Box highlights samples used for quantification.

Figure S3

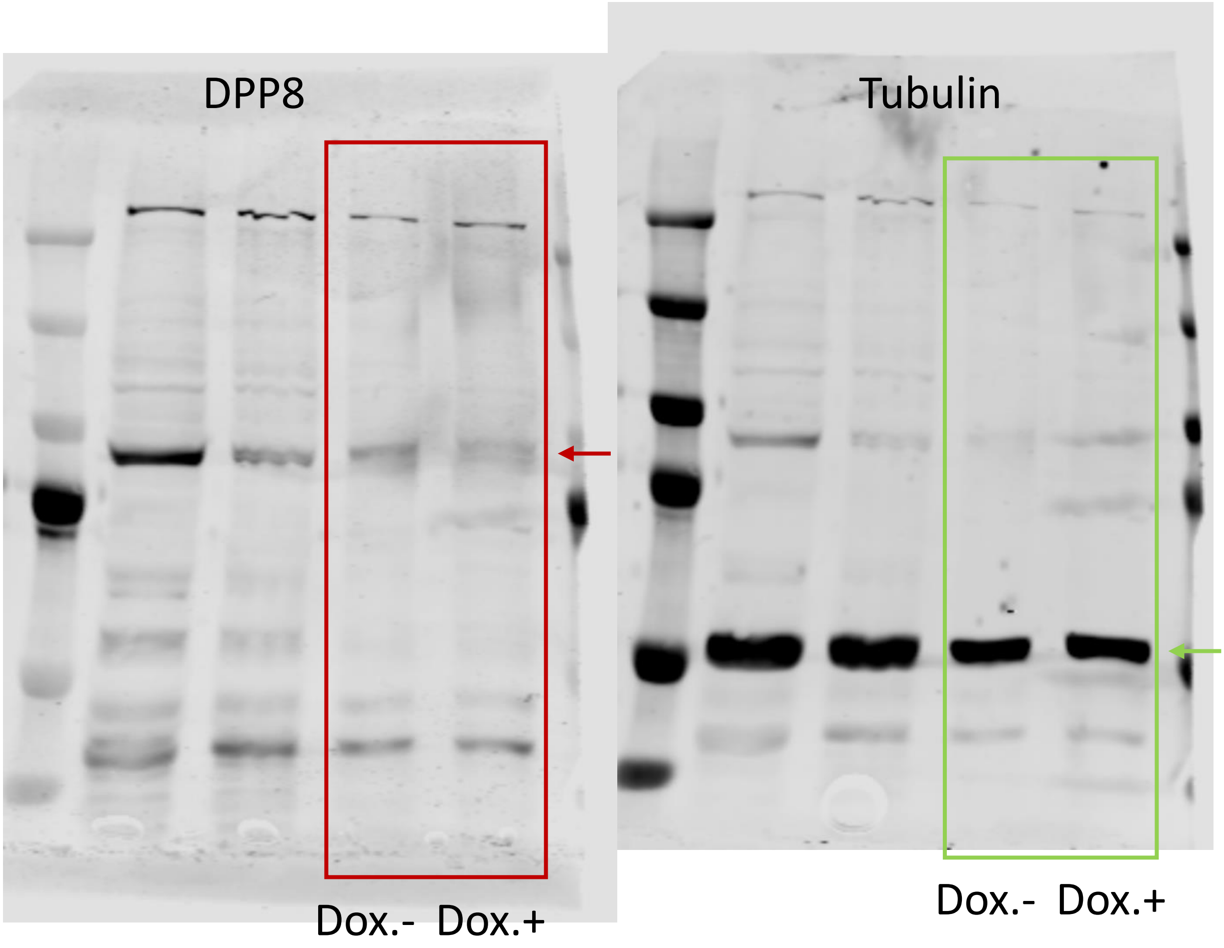

1 replicate is shown in the graph without quantification.
(Quantification of enzyme activity is shown)
Box highlights samples used for quantification.
